# PDS5A and PDS5B differentially affect gene expression without altering cohesin localization across the genome

**DOI:** 10.1186/s13072-022-00463-6

**Published:** 2022-08-19

**Authors:** Nicole L. Arruda, Audra F. Bryan, Jill M. Dowen

**Affiliations:** 1 Curriculum in Genetics and Molecular Biology, University of North Carolina at Chapel Hill, Chapel Hill, NC 27599 USA; 2 Integrative Program for Biological and Genome Sciences, University of North Carolina at Chapel Hill, Chapel Hill, NC 27599 USA; 3 Department of Biochemistry and Biophysics, University of North Carolina at Chapel Hill, Chapel Hill, NC 27599 USA; 4 Department of Biology, University of North Carolina at Chapel Hill, Chapel Hill, NC 27599 USA; 5 Lineberger Comprehensive Cancer Center, University of North Carolina at Chapel Hill, Chapel Hill, NC 27599 USA

**Keywords:** Cohesin, Genome, Localization, Gene expression, PDS5A, PDS5B

## Abstract

**Background:**

Cohesin is an important structural regulator of the genome, regulating both three-dimensional genome organization and gene expression. The core cohesin trimer interacts with various HEAT repeat accessory subunits, yielding cohesin complexes of distinct compositions and potentially distinct functions. The roles of the two mutually exclusive HEAT repeat subunits PDS5A and PDS5B are not well understood.

**Results:**

Here, we determine that PDS5A and PDS5B have highly similar localization patterns across the mouse embryonic stem cell (mESC) genome and they show a strong overlap with other cohesin HEAT repeat accessory subunits, STAG1 and STAG2. Using CRISPR/Cas9 genome editing to generate individual stable knockout lines for PDS5A and PDS5B, we find that loss of one PDS5 subunit does not alter the distribution of the other PDS5 subunit, nor the core cohesin complex. Both PDS5A and PDS5B are required for proper gene expression, yet they display only partially overlapping effects on gene targets. Remarkably, gene expression following dual depletion of the PDS5 HEAT repeat proteins does not completely overlap the gene expression changes caused by dual depletion of the STAG HEAT repeat proteins, despite the overlapping genomic distribution of all four proteins. Furthermore, dual loss of PDS5A and PDS5B decreases cohesin association with NIPBL and WAPL, reduces SMC3 acetylation, and does not alter overall levels of cohesin on the genome.

**Conclusions:**

This work reveals the importance of PDS5A and PDS5B for proper cohesin function. Loss of either subunit has little effect on cohesin localization across the genome yet PDS5A and PDS5B are differentially required for gene expression.

**Supplementary Information:**

The online version contains supplementary material available at 10.1186/s13072-022-00463-6.

## Background

The cohesin complex dynamically structures chromosomes throughout the cell cycle, mediating DNA loops during interphase and maintaining sister chromatid cohesion after DNA replication until mitosis [1, 2]. Cohesin consists of three core subunits, SMC1A, SMC3, and RAD21, and interacts with a variety of HEAT repeat proteins that contribute to distinct cohesin functions. For example, cohesin has mutually exclusive interactions with one of two HEAT repeat accessory subunits, STAG1 or STAG2, when it binds to DNA and forms a DNA loop [3–6]. The precocious dissociation of sisters 5 (PDS5) subunits, PDS5A and PDS5B, are another pair of mutually exclusive HEAT repeat accessory subunits that bind cohesin–STAG1/2 complexes. PDS5 proteins have important roles in sister chromatid cohesion, as PDS5 loss results in aberrant sister chromatid arrangement, disrupts development, and leads to embryonic lethality [7–11]. However, the roles of PDS5A and PDS5B subunits in cohesin-mediated genome organization and gene expression are not well understood. Furthermore, the relationship between PDS5 proteins and STAG1 or STAG2 is not clear. While STAG1 and STAG2 have been shown to localize to mostly shared binding sites across the genome, the localization pattern of PDS5A and PDS5B is not known [12–15]. It is possible that cohesin complexes of distinct compositions (ex. cohesin–STAG1–PDS5A versus cohesin–STAG2–PDS5B) have specific roles or properties that differentially impact genome structure and function.

The interaction of PDS5 proteins with the cohesin complex is thought to be mutually exclusive with that of the cohesin loading factor NIPBL, another HEAT repeat protein that is critical for cohesin loading onto DNA and extrusion of DNA loops [4–6, 16–18]. Current models suggest that NIPBL helps cohesin complexes load onto chromosomes and extrude DNA until distal sites are encountered where NIPBL dissociates and/or is displaced by PDS5A or PDS5B. PDS5 subunits have recently been hypothesized to interact with CTCF and participate in the capture and stable association of cohesin complexes at the anchors of DNA loops [19, 20]. However, it is unclear how cohesin is stabilized at sites that lack CTCF, such as enhancers and promoters [19–24]. PDS5A and PDS5B are reported to function in chromatin looping, replication fork protection, and WAPL-mediated unloading of cohesin from DNA during interphase [20, 25–28]. Regarding genome organization, the simultaneous depletion of both PDS5A and PDS5B in HeLa cells results in a reduced number of DNA loops and weakens the strength of topologically associating domains (TADs) and compartments [20]. Furthermore, loss of PDS5A and PDS5B results in fewer loops with convergently oriented CTCF DNA motifs and more loops with divergent and/or tandemly oriented CTCF DNA motifs [20].

Cohesin-mediated DNA loops are a driving force in genome structure, despite their transient nature. Recent data suggest that DNA loops are relatively rare, forming < 7% of the time and lasting < 30 min [29]. It is not clear how cohesin-mediated DNA extrusion is halted, causing dynamic cohesin molecules to be stabilized at specific sites. Some reports indicate that acetylation of the cohesin subunit SMC3 (SMC3ac) at residues K105 and K106 in mammals (K112 and K113 in yeast) enables stable cohesin binding, though it remains unclear whether SMC3 acetylation is important for the stability of DNA loops that regulate gene expression [30–32]. During S phase, the acetyltransferases ESCO1 and ESCO2 are responsible for SMC3-K105/6 acetylation [33, 34]. While expression of ESCO2 is limited to S phase, expression of ESCO1, and subsequently the acetylation of SMC3, is prevalent throughout the cell cycle [32, 35–37]. In yeast, SMC3 mutant strains that cannot be acetylated due to mutation of the lysine residues are inviable, while the equivalent mutations in mammalian SMC3 are viable [33, 38, 39]. In addition, while cohesin residency time on chromatin does not change in SMC3ac mutant mammalian cells, yeast SMC3ac mutant cells show reduced cohesin binding, causing a decrease in DNA loop number and a shift toward larger DNA loops [33, 38, 39]. SMC3 acetylation is reduced to varying degrees upon loss of PDS5A, PDS5B, STAG1, STAG2, or CTCF [11, 32, 37, 40–43]. The mechanisms underlying how these various cohesin interacting proteins can all contribute to the acetylation status of SMC3 is unknown.

In this study, we utilize CRISPR/Cas9 genome editing, genomics techniques, and biochemical assays to investigate the roles of PDS5A and PDS5B in mouse embryonic stem cells. We find that PDS5A and PDS5B localize to shared binding sites across the genome and do not regulate the location of cohesin binding to chromatin. Despite displaying a shared pattern of localization across the genome, PDS5A and PDS5B have partially distinct effects on gene expression. A comparative analysis of all four HEAT repeat accessory proteins reveals that PDS5A/B and STAG1/2 localize to shared sites across the genome and cause similar decreases in SMC3 acetylation following dual loss of PDS5 subunits or STAG subunits. Dual loss of both PDS5 subunits reduces the interaction between cohesin and its known regulators NIPBL and WAPL, while dual loss of both STAG proteins decreases the levels of chromatin-bound cohesin. Together these data reveal a requirement for PDS5 subunits in proper gene expression but not cohesin localization or levels on the genome.

## Results

### PDS5A and PDS5B localize to shared binding sites across the genome

**Fig. 1:**
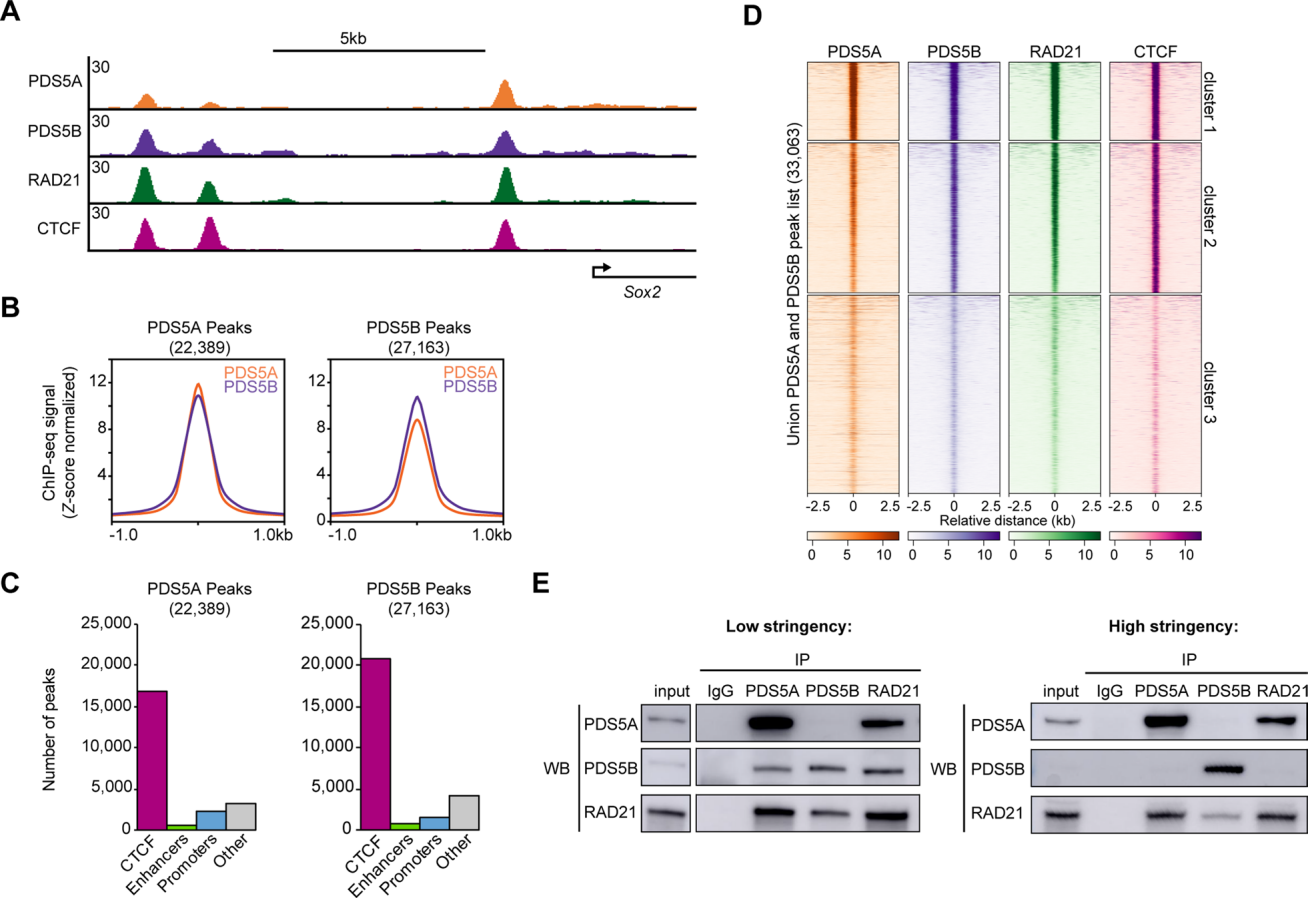
PDS5A and PDS5B localize to shared sites across the mESC genome. **A** Genome browser tracks of PDS5A, PDS5B, RAD21, and CTCF ChIP-seq signal near the promoter of *Sox2* in WT mESCs (*Z-*score normalized). **B **Average signal plots of PDS5A and PDS5B ChIP-seq signal at all PDS5A peaks and all PDS5B peaks (*Z-*score normalized). **C** Number of PDS5A and PDS5B peaks overlapping relevant functional elements across the genome: CTCF sites, enhancers, promoters, or other (none of the above). **D** Clustered heatmaps of PDS5A, PDS5B, RAD21, and CTCF ChIP-seq signal at a union list of PDS5A and PDS5B peaks, ordered by RAD21 signal (k-means = 3) (*Z-*score normalized). **E** Western blot analysis following co-immunoprecipitation of IgG (negative control), PDS5A, PDS5B, and RAD21 in WT nuclear lysates, under low and high stringency conditions

To determine the genome-wide occupancy patterns of PDS5A and PDS5B, chromatin immunoprecipitation followed by high-throughput sequencing (ChIP-seq) was performed in mouse embryonic stem cells (mESCs), utilizing a spike-in for normalization (Additional file 1: Table S1). PDS5A and PDS5B displayed similar binding profiles across the genome and had a similar number of peaks, with 22,389 PDS5A peaks and 27,163 PDS5B peaks identified (Fig. 1A, B, Additional file 2: Fig. S1A, B). PDS5A and PDS5B signal showed strong overlap with signal for the core cohesin subunit, RAD21, and CTCF. PDS5A and PDS5B peaks showed strong overlap with CTCF sites and relatively little overlap with enhancers or promoters (Fig. 1C). A union peak list of all PDS5A peaks and all PDS5B peaks (33,063) was subjected to k-means clustering and showed that sites of strong PDS5A signal also display strong signal for PDS5B, RAD21, and CTCF (Fig. 1D). Though PDS5A and PDS5B localized to a shared set of genomic sites in a population of cells, it was not clear whether they co-occupied a single site at the same time. Co-immunoprecipitation (coIP) experiments revealed that immunoprecipitation of PDS5A was able to co-purify RAD21 and PDS5B under low stringency, uncrosslinked conditions (Fig. 1E). However, immunoprecipitation of PDS5B only co-purified the core cohesin subunit RAD21 and not PDS5A. When these coIPs were performed under high stringency (high salt and detergent), uncrosslinked conditions, the interaction between PDS5A and PDS5B was no longer detectable, yet the interaction between PDS5A and the core cohesin subunit RAD21 remained intact. Interestingly, under high stringency conditions PDS5B showed decreased interaction with the core cohesin complex, suggesting that it is more weakly associated with the cohesin ring than PDS5A. These results indicate that individual cohesin complex molecules contain one of the two mutually exclusive PDS5 subunits. While under low stringency conditions it is possible to detect weak interactions between PDS5A and PDS5B, this likely reflects the weak interactions that can occur between two individual variant cohesin complexes (a cohesin–PDS5A complex interacting with a cohesin–PDS5B complex). Together, these results suggest that while PDS5A and PDS5B occupy many shared sites across the genome, they participate in distinct cohesin complex molecules and are not part of a stable higher order structure of multiple cohesin molecules.

### Loss of one PDS5 subunit does not impact localization of cohesin, nor the other PDS5 subunit

**Fig. 2:**
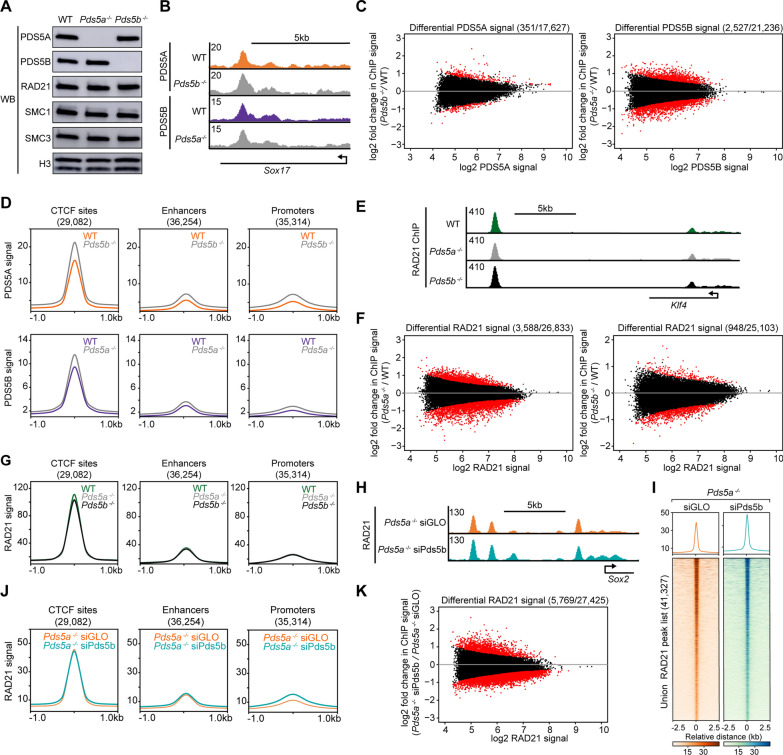
Loss of a PDS5 protein does not cause mislocalization of the other PDS5 protein or the cohesin complex. **A** Western blot analysis of cohesin complex subunits in WT, *Pds5a*^*−/−*^, and *Pds5b*^*−/−*^ mESC nuclear lysates. **B** Genome browser tracks of PDS5A ChIP-seq signal in WT and *Pds5b*^*−/−*^ mESCs and PDS5B signal in WT and *Pds5a*^*−/−*^ mESCs at the *Sox17* gene locus. **C** MA plots showing differential enrichment of PDS5A signal between WT and *Pds5b*^*−/−*^ mESCs at conserved binding sites, as well as differential enrichment of PDS5B signal between WT and *Pds5a*^*−/−*^ mESCs at conserved binding sites. **D** Average signal plots of PDS5A ChIP-seq signal in WT and *Pds5b*^*−/−*^ mESCs, as well as PDS5B signal in WT and *Pds5a*^*−/−*^ mESCs, at CTCF sites, enhancers, and promoters. **E** Genome browser tracks of RAD21 ChIP-seq signal in WT, *Pds5a*^*−/−*^, and *Pds5b*^*−/−*^ mESCs at the *Klf4* gene locus. **F** MA plots showing differential enrichment of RAD21 signal between WT and *Pds5a*^*−/*−^ mESCs at conserved binding sites, as well between WT and *Pds5b*^*−/−*^ mESCs at conserved binding sites. **G** Average signal plots of RAD21 ChIP-seq signal in WT, *Pds5a*^*−/−*^, and *Pds5b*^*−/−*^ mESCs at CTCF sites, enhancers, and promoters. **H** Genome browser tracks of RAD21 ChIP-seq signal in *Pds5a*^*−/−*^ siGLO and *Pds5a*^*−/−*^ siPds5b mESCs at the *Sox2* gene locus. **I** Heatmaps of RAD21 ChIP-seq signal in *Pds5a*^*−/−*^ siGLO and *Pds5a*^*−/−*^ siPds5b mESCs at a union peak list of all RAD21 peaks in both conditions. **J** Average signal plots of RAD21 ChIP-seq signal in *Pds5a*^*−/−*^ siGLO and *Pds5a*^*−/−*^ siPds5b mESCs at CTCF sites, enhancers, and promoters. **K** MA plot showing differential RAD21 ChIP-seq signal between *Pds5a*^*−/−*^ siPds5b and *Pds5a*^*−/−*^ siGLO mESCs at conserved binding sites

To investigate the individual roles of PDS5A and PDS5B in cohesin biology, CRISPR/Cas9 genome editing was used to generate two independent knockout mESC lines for each gene: *Pds5a*^*−/−*^ rep 1, *Pds5a*^*−/−*^ rep 2, *Pds5b*^*−/−*^ rep 1, and *Pds5b*^*−/−*^ rep 2 mESCs (Additional file 2: Fig. S2A). Notably, PDS5A protein was undetectable in *Pds5a*^*−/−*^ mESCs and PDS5B protein was undetectable in *Pds5b *^*−/−*^ mESCs, and, the levels of other cohesin subunits were not altered (Fig. 2A). To determine if one PDS5 subunit compensates for the loss of the other PDS5 subunit, ChIP-seq was performed, with a spike-in for normalization, for PDS5A in wildtype (WT) and *Pds5b*^*−/−*^ mESCs, and for PDS5B in WT and *Pds5a *^*−/−*^ mESCs. Importantly, PDS5A and PDS5B immunoprecipitation efficiencies in WT and knockout mESCs were similar for all replicates (Additional file 2: Fig. S2B, C). The PDS5A binding profile was similar in WT and *Pds5b*^*−/−*^ mESCs (with 22,389 and 22,022 peaks identified, respectively), and the binding profile of PDS5B was very similar in WT and *Pds5a*^*−/−*^ mESCs (with 27,163 and 27,088 peaks identified, respectively) (Fig. 2B, Additional file 2: Fig. S2D). Analysis of relative signal was performed with DiffBind (which measures quantitative changes at binding sites shared by two conditions) [44], and revealed a small percentage of sites with differential ChIP-seq signal for PDS5A in *Pds5b*^*−/−*^ mESCs relative to WT (2.0%), and PDS5B in *Pds5a*^*−/−*^ mESCs relative to WT (11.9%) (Fig. 2C). At CTCF sites, enhancers, and promoters, PDS5A levels were largely unaltered by loss of PDS5B, and PDS5B levels were largely unaltered by loss of PDS5A (Fig. 2D). This result, along with the small percentages of differential binding across the genome, indicates that the PDS5 proteins do not compensate for one another. Following the chronic loss of one PDS5 subunit, there is not a major redistribution of the remaining PDS5 subunit to new genomic sites, nor altered levels of the remaining PDS5 subunit at conserved binding sites.

While the localization patterns of PDS5A and PDS5B were largely insensitive to loss of one another, it remained unclear if localization of the core cohesin complex was also insensitive to loss of either PDS5 subunit. To address this, ChIP-seq for RAD21 in WT, *Pds5a*^*−/−*^*,* and *Pds5b*^*−/−*^ mESCs was performed, and notably, similar ChIP efficiencies were observed for all replicates (Additional file 2: Fig. S2E). The results revealed similar binding profiles and peak numbers for RAD21 in WT, *Pds5a*^*−/−*^*,* and *Pds5b*^*−/−*^ mESCs (33,665, 35,981, and 31,595 peaks, respectively) (Fig. 2E, Additional file 2: Fig. S2F). Consistent with this result, DiffBind analysis detected relatively few sites of differential RAD21 signal in *Pds5a*^*−/−*^ or *Pds5b*^*−/−*^ mESCs relative to WT (13.4% and 3.8%, respectively), indicating that levels of the core cohesin complex across the genome display minor changes following loss of either PDS5A or PDS5B (Fig. 2F). RAD21 signal at CTCF sites, enhancers, and promoters was also strikingly similar across all three cell lines (Fig. 2G). These results indicate that PDS5A and PDS5B subunits are not specificity factors that dictate where cohesin localizes on the genome and that cohesin complexes lacking PDS5 subunits are still distributed to their normal genomic sites. Furthermore, there were no ectopic PDS5 binding sites nor ectopic RAD21 binding sites observed in cells lacking a single PDS5 protein, consistent with the model that PDS5 subunits are mostly in complex with the core cohesin members and do not operate independently of the core complex.

### Dual loss of PDS5A and PDS5B does not alter cohesin localization or levels on the genome

To investigate whether simultaneous loss of both PDS5A and PDS5B impacts cohesin localization on chromatin, siRNA knockdown of PDS5B was performed in *Pds5a*^*−/−*^ mESCs. As controls, siPds5b and siGLO transfections were also performed in WT mESCs. Upon siRNA treatment in *Pds5a*^*−/−*^ mESCs, PDS5B protein levels were depleted by 95%, relative to levels in siGLO control treated *Pds5a*^*−/−*^ mESCs (Additional file 2: Fig. S2G). RAD21 ChIP-seq was performed in the four siRNA conditions, employing a spike-in for normalization (Fig. 2H, Additional file 2: Fig. S2H, I). Knockdown of PDS5B in either WT or *Pds5a*^*−/−*^ mESCs did not alter cohesin localization at a union list of RAD21 peaks identified in either condition (Fig. 2I, Additional file 2: Fig. S2J). The levels of cohesin at CTCF sites, enhancers, and promoters in the siPds5b treated cells were unchanged, compared to controls (Fig. 2J, Additional file 2: Fig. S2K). Depletion of PDS5B caused a significant change in RAD21 levels at 21% of cohesin binding sites in *Pds5a*^*−/−*^ mESCs, which was similar to the effect of depletion of PDS5B in WT mESCs (20.4% of sites displayed differential signal), suggesting that acute loss of PDS5B in WT cells or dual loss of both PDS5B and PDS5A caused similar, minor changes in cohesin levels at conserved binding sites (Fig. 2K, Additional file 2: Fig. S2L). These results show that cohesin localizes normally in the absence of PDS5 proteins, suggesting that PDS5A and PDS5B are not specificity factors that dictate the sites of cohesin binding on the genome.

### PDS5A and PDS5B have both overlapping and distinct effects on gene expression

**Fig. 3:**
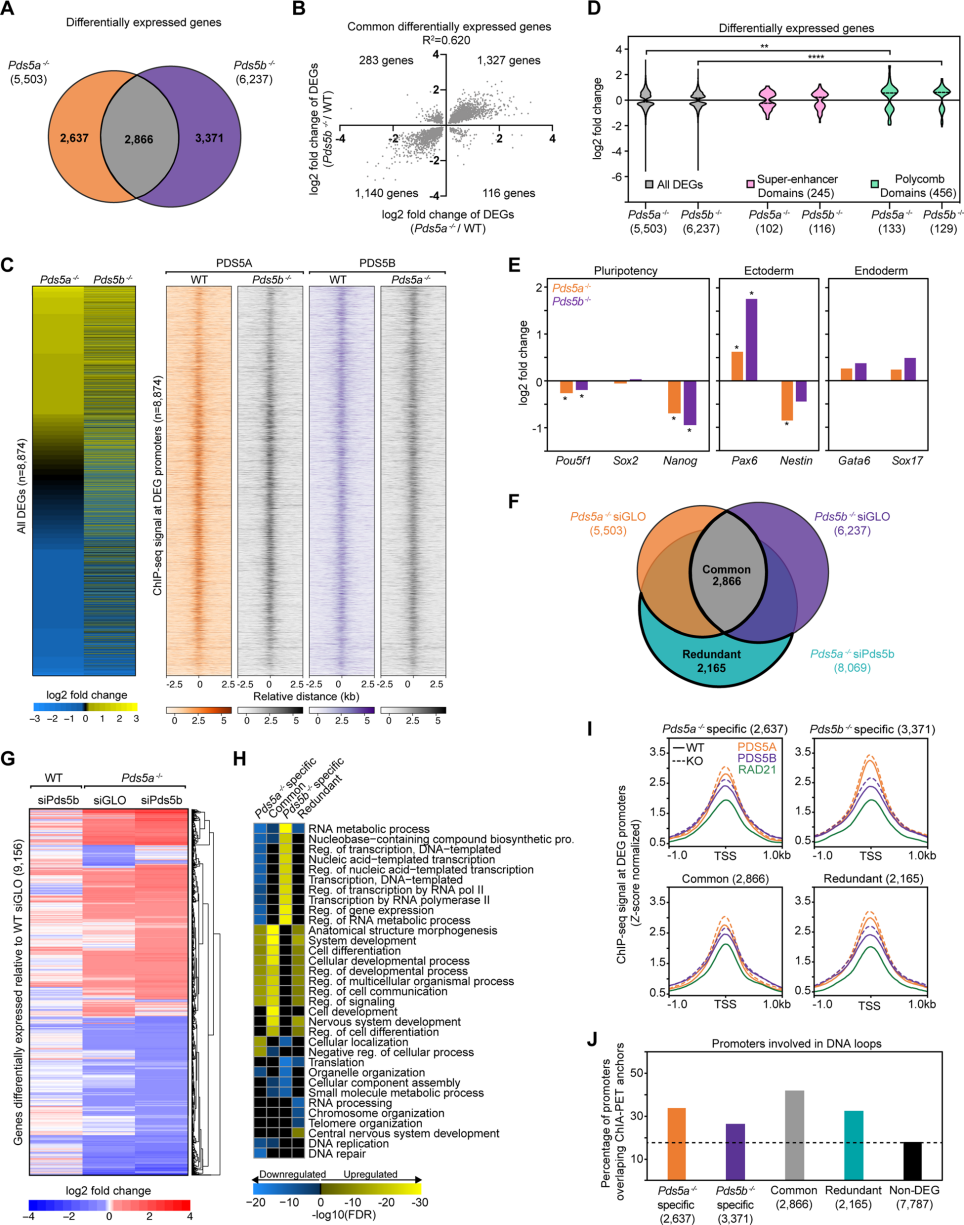
PDS5A and PDS5B display partially overlapping and distinct effects on gene expression**. A** Overlap of differentially expressed genes (DEGs) in *Pds5a*^*−/−*^ and *Pds5b*^*−/−*^ mESCs relative to WT mESCs. All cells treated with siGLO as a transfection control. **B** Correlation plot of log2 fold change in expression of Common DEGs (2866) in *Pds5a*^*−/−*^ and *Pds5b*^*−/−*^ mESCs. Axes cropped, removing one outlier. **C** Heatmap of log2 fold change in expression for all DEGs in both *Pds5a*^*−/−*^ and *Pds5b*^*−/−*^ mESCs. ChIP-seq signal of PDS5A in WT and *Pds5b*^*−/−*^ mESCs and PDS5B signal in WT and *Pds5a*^*−/−*^ mESCs is shown at the promoters of these DEGs. **D** Violin plot of log2 fold change in expression for all DEGs, those within Super-enhancer Domains, and those within Polycomb Domains in *Pds5a*^*−/−*^ and *Pds5b*^*−/−*^ mESCs. Significance was determined using a Kruskal–Wallis test followed by Dunn’s multiple comparisons test. Asterisks indicate significant differences between groups (** *p* < 0.01, **** *p* < 0.0001). **E** Bar graphs of log2 fold change in expression of pluripotency genes (*Pou5f1, Sox2, Nanog*), ectodermal lineage genes (*Pax6* and *Nestin*), and endodermal lineage genes (*Gata6* and *Sox17*) in *Pds5a*^*−/−*^ and *Pds5b*^*−/−*^ mESCs, relative to WT mESCs. Asterisks indicate significant differences from WT determined using DESeq2 (padj < 0.01). **F** Overlap of differentially expressed genes in *Pds5a*^*−/−*^ siGLO, *Pds5b*^*−/−*^ siGLO, and *Pds5a*^*−/−*^ siPds5b mESCs. Common and Redundant gene classes are highlighted. **G** Clustered heatmap of log2 fold change in expression for a combined list of DEGs in WT siPds5b, *Pds5a*^*−/−*^ siGLO, and *Pds5a*^*−/−*^ siPds5b mESCs all relative to WT siGLO mESCs. **H** Gene Ontology (GO) analysis for biological processes correlated with DEGs that are *Pds5a*^*−/−*^ specific, *Pds5b*^*−/−*^ specific, Common, or Redundant upon loss of PDS5 proteins. **I** Average signal plots of PDS5A, PDS5B, and RAD21 ChIP-seq signal in WT (solid line) and knockout (dotted) mESCs at *Pds5a*^*−/−*^ specific, *Pds5b*^*−/−*^ specific, Common, or Redundant DEG promoters (*Z-*score normalized). **J** Percentages of promoters in *Pds5a*^*−/−*^ specific, *Pds5b*^*−/−*^ specific, Common, Redundant, or Non-DEG gene classes that overlap with WT cohesin ChIA–PET high-confidence interaction anchors [46]

Whether PDS5 subunits play a role in regulating gene expression is unclear. To investigate this, RNA-seq was performed in WT, *Pds5a*^*−/−*^*, *and* Pds5b*^*−/−*^ mESCs and differential gene expression analysis was performed using DESeq2 (padj < 0.01) [45]. *Pds5a*^*−/−*^ mESCs had 5,503 differentially expressed genes (DEGs) compared to WT mESCs, and *Pds5b*^*−/−*^ mESCs had 6,237 DEGs compared to WT mESCs (Additional file 1: Table S1, Additional file 2: Fig. S3A, Additional file 3: Table S2). An overlap of these DEG lists revealed 2,866 genes that were misexpressed upon loss of either PDS5A or PDS5B (Common), while 2,637 genes were misexpressed only in *Pds5a*^*−/−*^ mESCs and 3,371 genes were misexpressed only in *Pds5b*^*−/−*^ mESCs (Fig. 3A). Changes in gene expression of all DEGs in either *Pds5a*^*−/−*^ mESCs or *Pds5b*^*−/−*^ mESCs were weakly positively correlated (*R*^*2*^ = 0.359); however, there was a strong correlation at the 2,866 Common DEGs (*R*^*2*^ = 0.620) (Fig. 3B, Additional file 2: Fig. S3B). A combined list of all DEGs in *Pds5a*^*−/−*^ and *Pds5b*^*−/−*^ mESCs (8,874 genes) revealed similar patterns of log2 fold change in expression for both genotypes (Fig. 3C). Importantly, both PDS5A and PDS5B occupied the promoters of these genes and the relative ChIP-seq signal for PDS5A and PDS5B did not change at these promoters in the reciprocal knockout cell lines compared to WT (Fig. 3C). Gene expression within Super-enhancer Domains (SDs) and Polycomb Domains (PDs) was examined, since DNA loop structures at these domains are known to contain cell identity genes regulated by super-enhancers or repressed by Polycomb, and as such these genes are often sensitive to loss of transcriptional insulation [46]. Expression of genes within PDs significantly increased in both *Pds5a*^*−/−*^ and *Pds5b*^*−/−*^ mESCs (*p* < 0.01), while expression of genes within SDs did not change (Fig. 3D). To examine whether loss of PDS5A or PDS5B impacted cellular identity, the expression of pluripotency, ectoderm, and endoderm genes was examined. *Pds5a*^*−/−*^ and *Pds5b*^*−/−*^ mESCs displayed significant decreases in expression of the pluripotency genes *Pou5f1* (OCT4) and *Nanog*, as well as increased expression of the ectodermal gene *Pax6*, consistent with altered stem cell state (Fig. 3E). PDS5A and PDS5B protein abundance is highly similar across a wide variety of cell lines and tissues [47], and while PDS5A transcript levels are higher than PDS5B in WT mESCs, loss of one of the PDS5 subunits did not significantly change the transcript level of the remaining PDS5 subunit (Fig. 2A, Additional file 2: Figure S3C–E).

### Redundant roles for PDS5A and PDS5B in expression of a set of genes

To further investigate potential overlapping roles of PDS5A and PDS5B in regulating gene expression, RNA-seq was performed in *Pds5a*^*−/−*^ siPds5b mESCs and three control conditions. Knockdown of PDS5B in *Pds5a*^−/−^ mESCs resulted in a large number of DEGs relative to WT siGLO treated mESCs, while PDS5B knockdown in WT mESCs caused virtually no genes to change in expression (8,069 versus 16, respectively) (Additional file 2: Figure S3F). An overlap of DEGs in *Pds5a*^*−/−*^ siPds5b mESCs with those in *Pds5a*^*−/−*^ siGLO and *Pds5b*^*−/−*^ siGLO mESCs revealed a class of genes, where PDS5A and PDS5B act redundantly (2,165 genes) (Fig. 3F). In addition, this analysis showed that dual depletion of both PDS5 subunits created more DEGs than either single perturbation condition. Genes that were considered differentially expressed in any condition compared to the WT siGLO condition were further analyzed. The log2 fold change in gene expression was stronger overall in the dual depletion condition than either single condition (Fig. 3G). Similar to both single knockout lines, dual depletion of both PDS5 subunits caused significant upregulation of genes within PDs (*p* < 0.0001); however, dual depletion also led to a significant decrease in expression of genes within SDs, which was not observed in *Pds5a*^*−/−*^ or *Pds5b*^*−/−*^ mESCs (*p* < 0.0001) (Additional file 2: Figure S3G). Changes to pluripotency and ectodermal genes were detected in the *Pds5a*^*−/−*^ siPds5b mESCs, with a similar direction of change but a greater fold change than in *Pds5a*^*−/−*^ and *Pds5b*^*−/−*^ mESCs (Additional file 2: Figure S3H). Gene ontology (GO) analysis revealed strong differences in the biological processes linked to *Pds5a*^*−/−*^ specific and *Pds5b*^*−/−*^ specific DEGs (Fig. 3H, Additional file 4: Table S3). In general, the biological processes altered in the Common, Redundant, and *Pds5a*^*−/−*^ specific classes were similar to one another, while the *Pds5b*^*−/−*^ specific class of genes showed a more distinct pattern (Fig. 3H). Overall, the genes at which PDS5A and PDS5B act redundantly are implicated in neuronal differentiation, neuronal generation, and RNA processing. Although some genes were commonly, redundantly, or specifically affected by loss of either PDS5A or PDS5B subunits, there was no specific enrichment for either PDS5A or PDS5B occupancy at the promoters of these four gene classes (Fig. 3I). Furthermore, there were no changes in PDS5A or PDS5B enrichment in *Pds5b*^*−/−*^ or *Pds5a*^*−/−*^ cells, respectively, compared to WT mESCs, indicating that these gene expression changes may be an indirect effect (Fig. 3I). Notably, promoters of genes differentially expressed in these four gene classes were more likely to be engaged in a long-range interaction (26–42%) than promoters of genes not differentially expressed following PDS5 perturbations (18%) (Fig. 3J) [46]. Taken together, these results reveal that PDS5A and PDS5B act redundantly to preserve proper expression of a subset of genes while also displaying distinct effects on other subsets of genes.

### PDS5 subunits and STAG subunits localize to the same sites but have partially distinct effects on gene expression

**Fig. 4:**
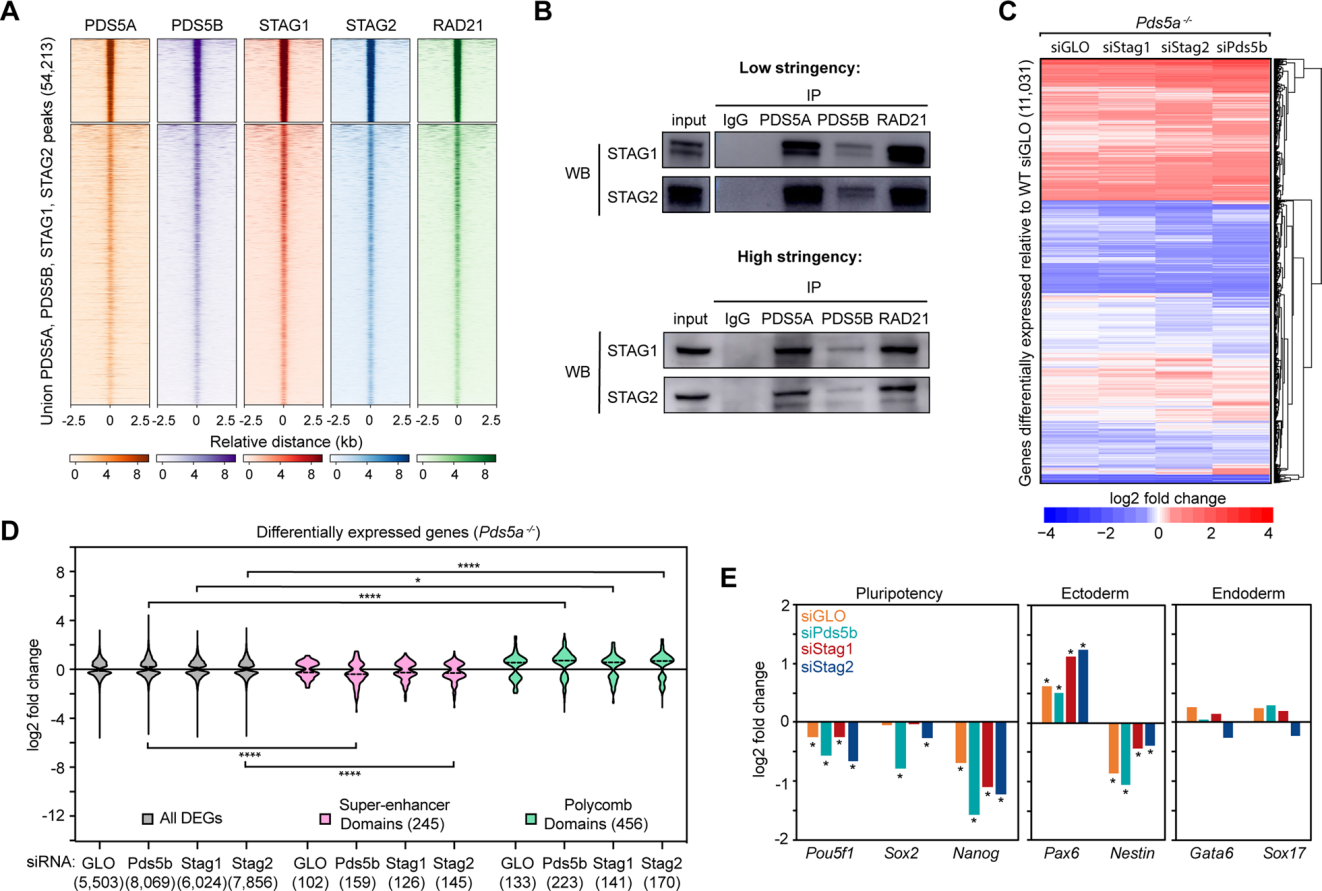
PDS5 proteins localize to the same genomic sites as STAG proteins**. A** Clustered heatmaps of PDS5A, PDS5B, STAG1, STAG2, and RAD21 ChIP-seq signal at a union list of PDS5A, PDS5B, STAG1, and STAG2 peaks, ordered by RAD21 signal (k-means = 2) (*Z-*score normalized). **B** Western blot analysis following co-immunoprecipitation of IgG (negative control), PDS5A, PDS5B, and RAD21 in WT nuclear lysates, under low and high stringency conditions. Control blots for this experiment are in Fig. 1E. **C** Clustered heatmap of log2 fold change in expression for a combined list of DEGs in *Pds5a*^*−/−*^ siGLO, *Pds5a*^*−/−*^ siStag1, *Pds5a*^*−/−*^ siStag2, and *Pds5a*^*−/−*^ siPds5b mESCs relative to WT siGLO mESCs. **D** Violin plot of log2 fold change in expression for all DEGs, those within Super-enhancer Domains, and those within Polycomb Domains for *Pds5a*^*−/−*^ siGLO, *Pds5a*^*−/−*^ siStag1, *Pds5a*^*−/−*^ siStag2, and *Pds5a*^*−/−*^ siPds5b mESCs relative to WT siGLO mESCs. Significance was determined using a Kruskal–Wallis test followed by Dunn’s multiple comparisons test. Asterisks indicate significant differences between groups (* *p* < 0.05, **** *p* < 0.0001). **E** Bar graphs of log2 fold change in expression of pluripotency genes (*Pou5f1, Sox2, Nanog*), ectodermal lineage genes (*Pax6 and Nestin*), and endodermal lineage genes (*Gata6 and Sox17*) in *Pds5a*^*−/−*^ siGLO, *Pds5a*^*−/−*^ siStag1, *Pds5a*^*−/−*^ siStag2, and *Pds5a*^*−/−*^ siPds5b mESCs. Asterisks indicate significant differences from WT siGLO mESCs determined using DESeq2 (padj < 0.01)

The discovery that PDS5A and PDS5B have both distinct and overlapping effects on gene expression despite their shared genome-wide occupancy is similar to previous results reported for the other set of mutually exclusive cohesin subunits, STAG1 and STAG2 [12]. However, the relationship between PDS5 proteins and STAG proteins has not yet been directly explored. Therefore, the genome-wide distribution of PDS5A, PDS5B, STAG1, and STAG2 was examined in WT mESCs and revealed a striking overlap of ChIP-seq signal for all four subunits at a union list including all peaks identified in any of the individual data sets (54,213) (Fig. 4A) [12]. Notably, the strongest PDS5 peaks are also the strongest STAG peaks, indicating that the chromatin-bound levels of all four subunits are positively correlated. Co-immunoprecipitation of PDS5A, PDS5B, and RAD21 was performed under both low and high stringency, uncrosslinked conditions, to investigate potential specificity in subunit composition of cohesin complexes; western blotting for STAG1 and STAG2 subunits demonstrated that STAG1 and STAG2 both co-purify with PDS5A and PDS5B subunits (Fig. 4B). This, together with our previous report demonstrating that STAG1 and STAG2 localize to shared genomic locations in distinct complexes, indicates that there is not a selective incorporation of either PDS5A or PDS5B with cohesin–STAG1 or cohesin–STAG2 variant complexes [12]. Rather, cohesin complexes of all possible compositions of STAG1 or STAG2 and PDS5A or PDS5B can exist in the cell. While it remained unclear whether the overlapping distribution of PDS5A/B and STAG1/2 on the genome was dependent on the presence of one another, ChIP–qPCR was performed for PDS5A and PDS5B in mESCs nearly devoid of STAG proteins, as well as ChIP–qPCR for STAG1 and STAG2 in mESCs nearly devoid of PDS5 proteins. ChIP enrichment for PDS5A and PDS5B was determined at individual CTCF binding sites in WT siGLO and *Stag2*^*−/−*^ siStag1 mESCs, and revealed no significant changes in PDS5 protein occupancy (Additional file 2: Figure S4A). ChIP enrichment for STAG1 and STAG2 was determined at the same CTCF binding sites in WT siGLO and *Pds5a*^*−/−*^ siPds5b mESCs, and revealed modestly decreased levels of STAG1 (*p* < 0.01), while STAG2 levels showed no change (Additional file 2: Figure S4B). Therefore, PDS5 and STAG protein localization appears to be largely independent of one another.

Whereas cohesin binding to the genome is greatly reduced in *Stag2*^*−/−*^ siStag1 mESCs [12], PDS5A and PDS5B showed no significant decreases at CTCF binding sites, suggesting that PDS5 proteins can interact with CTCF independent of the core cohesin complex [19, 20]. To directly test this hypothesis, ChIP–qPCR was performed for PDS5A and PDS5B at CTCF binding sites in WT siGLO and WT siSMC3 mESCs. While no significant differences in enrichment were observed for PDS5A, PDS5B was significantly decreased at all four CTCF sites following cohesin depletion (Additional file 2: Figure S4C). This supports a model, where PDS5A, but not PDS5B, is able to interact with CTCF independent of the core cohesin complex.

Though no preferential physical interactions were detected for PDS5A/B and STAG1/2 subunits by co-immunoprecipitation, suggesting that cohesin complexes of all possible subunit compositions can exist in the cell, it is not known if the PDS5 subunits and STAG subunits display overlapping effects on gene expression. To explore this possibility, RNA-seq was performed following knockdown of STAG1 or STAG2 in *Pds5a*^*−/−*^ mESCs (Additional file 3: Table S2). Differential gene expression analysis revealed a similar number of DEGs in *Pds5a*^*−/−*^ siStag1 and *Pds5a*^*−/−*^ siStag2 mESCs to that seen in *Pds5a*^*−/−*^ siPds5b mESCs (6,024, 7,856, and 8,069, respectively) (Additional file 2: Figure S4D–E). Notably, the number of DEGs in *Pds5a*^*−/−*^ siStag2 and *Pds5a*^*−/−*^ siPds5b mESCs was similar and increased relative to *Pds5a*^*−/−*^ mESCs (5,503) and *Pds5b*^*−/−*^ mESCs (6,237). Genes that were differentially expressed in any of the four conditions relative to WT siGLO (*Pds5a*^*−/−*^ siGLO, *Pds5a*^*−/−*^ siStag1, *Pds5a*^*−/−*^ siStag2, and *Pds5a*^*−/−*^ siPds5b) were identified and merged into a union list (11,031 genes). Examination of the log2 fold change values of DEGs in this list revealed minimal differences in cells, where STAG1, STAG2, or PDS5B were knocked down in the *Pds5a*^*−/−*^ background relative to the WT siGLO control treated cells (Fig. 4C). Furthermore, there was a strong overlap between the DEGs identified in *Pds5a*^*−/−*^ siPds5b, with the DEGs identified in *Pds5a*^*−/−*^ siStag1 and *Pds5a*^*−/−*^ siStag2 mESCs (Additional file 2: Figure S4E). The expression of genes within SDs was not affected in *Pds5a*^*−/−*^ siStag1 mESCs, but was significantly decreased in *Pds5a*^*−/−*^ siStag2 mESCs, consistent with previous findings that loss of STAG2, but not STAG1, affects expression of SD genes in the WT background (Fig. 4D) [12]. Depletion of STAG1 or STAG2 also caused changes in expression of pluripotency and ectodermal genes similarly, in both direction and magnitude, to depletion of PDS5B in *Pds5a*^*−/−*^ mESCs (Fig. 4E). To better examine the effect of individual knockdowns on gene expression in the *Pds5a*^*−/−*^ siGLO background, differentially expressed genes relative to *Pds5a*^*−/−*^ siGLO mESCs were identified (rather than relative to WT siGLO mESCs). This analysis revealed relatively fewer differentially expressed genes (380 in *Pds5a*^*−/−*^ siStag1, 2,698 in *Pds5a*^*−/−*^ siStag2, and 3,485 in *Pds5a*^*−/−*^ siPds5b mESCs) than when compared to the WT siGLO background, demonstrating that depletion of STAG1 or STAG2 in a *Pds5a*^*−/−*^ background caused transcriptional changes similar to depletion of PDS5B (Additional file 2: Figure S4F, G).

Though knockdown of a single STAG subunit in a PDS5 knockout background resembled dual loss of both PDS5A and PDS5B, about half of all DEGs identified in *Pds5a*^*−/−*^ siPds5b overlapped with those in *Stag2*^*−/−*^ siStag1 mESCs (Additional file 2: Figure S4H) [12]. Furthermore, there was even less overlap of commonly or redundantly regulated genes following dual loss of both PDS5 subunits versus following loss of both STAG subunits (Additional file 2: Figure S4I) [12]. There was also little overlap of the biological processes affected specifically by dual loss of PDS5 subunits or dual loss of STAG subunits (Additional file 2: Figure S4J, Additional file 4: Table S3). Interestingly, the genes commonly regulated by both dual depletion conditions that change expression in opposite directions do not produce any enrichment for a particular biological process. In conclusion, the PDS5A/B and STAG1/2 subunits show remarkable overlap in their localization pattern on the genome, yet dual loss of PDS5A and PDS5B, or dual loss of STAG1 and STAG2, impacted the expression of genes involved in distinct biological processes.

### PDS5 subunits and STAG subunits have differential effects on SMC3 acetylation and levels of cohesin on chromatin

**Fig. 5:**
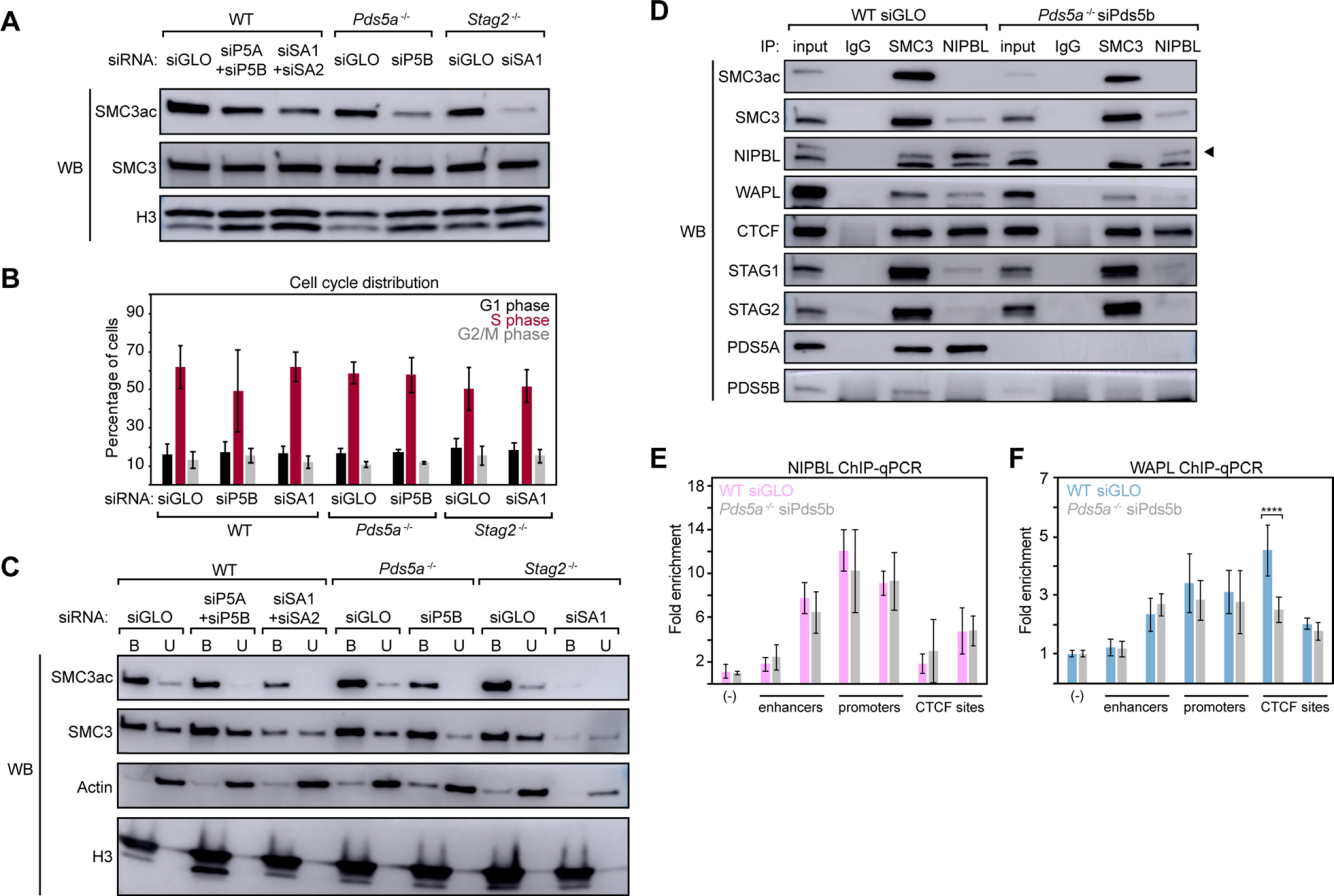
PDS5 proteins and STAG proteins have differential effects on SMC3ac levels and chromatin bound cohesin. **A** Western blot analysis of nuclear lysates from seven siRNA conditions in mESCs. Abbreviations included for PDS5A (P5A), PDS5B (P5B), STAG1 (SA1), and STAG2 (SA2). **B** Percentage of cells in each cell cycle phase, *n* = 3. A 2-tailed unpaired *t* test was used to determine significance. No significance was found between any mutant and WT condition for any cell cycle phase. **C** Western blot analysis following fractionation of cells from seven siRNA conditions. Both chromatin-bound (B) and nuclear soluble (U) are shown for each condition. **D** Western blot analysis following co-immunoprecipitation of IgG (negative control), SMC3, and NIPBL in WT siGLO and *Pds5a*^*−/−*^ siPds5b nuclear lysates, under high stringency conditions. Black triangle indicates the NIPBL-specific band (top), as determined via NIPBL knockdown (see Additional file 2: Figure S6D). **E** ChIP–qPCR for NIPBL in WT siGLO and *Pds5a*^*−/−*^ siPds5b mESCs. Fold enrichment relative to 5% input material and the negative control region is depicted. Data represented as mean ± standard deviation across two biological replicates, each with three technical replicates. A 2-tailed unpaired *t* test was used to determine significance between WT siGLO and *Pds5a*^*−/−*^ siPds5b mESCs for every primer set. No significance was found for any of the primer sets. **F** ChIP–qPCR for WAPL in WT siGLO and *Pds5a*^*−/−*^ siPds5b mESCs. Fold enrichment relative to 5% input material and the negative control region is depicted. Data represented as mean ± standard deviation across two biological replicates, each with three technical replicates. A 2-tailed unpaired *t* test was used to determine significance between WT siGLO and *Pds5a*^*−/−*^ siPds5b mESCs for every primer set. Asterisks indicate significant differences between groups (****p < 0.0001), otherwise, no significance was found

Given the many changes in gene expression following loss of PDS5A, PDS5B, STAG1, or STAG2 subunits, we next investigated various properties of the cohesin complex and its interactions with regulatory proteins. Initially, we sought to measure the post-translational acetylation of SMC3, at residues K105 and K106, associated with sister chromatid cohesion and stable cohesin binding during interphase [32–37]. Utilizing combinations of siRNA and knockout cell lines, 7 conditions were generated representing both acute (WT siPds5a + siPds5b and WT siStag1 + siStag2) and chronic (*Pds5a*^*−/−*^ siPds5b and *Stag2*^*−/−*^ siStag1) depletion of cohesin subunits (Additional file 2: Figure S5A). An SMC3 antibody and an SMC3ac-specific antibody (targeting SMC3 K105ac and K106ac) were used to detect levels of the post-translationally modified SMC3 and total SMC3 in nuclear extracts from the 7 cellular conditions. The results revealed decreased SMC3ac levels in all conditions compared to WT siGLO, with the strongest decreases observed in the dual depletion conditions: *Pds5a*^*−/−*^ siPds5b and *Stag2*^*−/−*^ siStag1 (Fig. 5A, Additional file 2: Figure S5B). Importantly, total SMC3 levels were unchanged across the 7 conditions and both antibodies were shown to be specific (Fig. 5A, Additional file 2: Figure S5C, D). The specific reduction in SMC3ac levels in *Pds5a*^*−/−*^ siPds5b mESCs compared to control mESCs was also observed following an IP of SMC3 in nuclear extracts (Additional file 2: Figure S5E). To address whether this loss of SMC3ac was due to a change in cell cycle distribution, the percentage of cells in G1, S, and G2/M was measured in dual depletion conditions as well as single knockdown controls. There were no significant differences in cell cycle distribution detected for any mutant condition relative to WT siGLO mESCs (Fig. 5B, Additional file 2: Figure S5F). Notably, *Pds5a*^*−/−*^ siPds5b mESCs showed a decrease in proliferation rate and *Stag2*^*−/−*^ siStag1 mESCs showed a strong proliferation defect and increased length of time in each cell cycle phase (Additional file 2: Figure S5G, H). These results indicate that reduced SMC3ac levels upon loss of PDS5 or STAG proteins are not due to a change in the proportion of cells in S phase or G2/M.

Subsequently, to address whether the decreases in SMC3ac impacted the levels of cohesin bound to the genome, we performed a chromatin fractionation in the same 7 conditions to assess the bound and unbound pools of cohesin. First, total SMC3 was found at similar levels in both the chromatin-bound and unbound fractions for all conditions except the *Stag2*^*−/−*^ siStag1 condition, consistent with our previous observation that cohesin is strongly lost from the genome in the absence of both STAG1 and STAG2 (Fig. 5C, Additional file 2: Figure S6A) [12]. Notably, both acute and chronic dual depletion of STAG1 and STAG2 subunits resulted in decreased SMC3 levels bound to chromatin. Given the reported role of PDS5 proteins in facilitating WAPL-mediated unloading of cohesin from the genome, it was surprising to see that the levels of bound cohesin were unchanged rather than increased following depletion of the PDS5 subunits (Additional file 2: Figure S6A). Second, SMC3ac levels decreased in both bound and unbound fractions following acute and chronic dual depletions of PDS5 subunits and STAG subunits (Fig. 5C, Additional file 2: Figure S6B). Overall, SMC3ac levels were reduced upon loss of either pair of HEAT repeat proteins: PDS5A/B or STAG1/2.

### Role of PDS5A and PDS5B in cohesin complex interactions with regulators NIPBL and WAPL

To investigate how dual loss of PDS5A and PDS5B affects SMC3ac levels without impacting the levels of cohesin bound to chromatin, coIPs were performed in WT siGLO and *Pds5a*^*−/−*^ siPds5b mESCs and alterations in cohesin interactions were assessed. Immunoprecipitation of SMC3 following dual loss of PDS5 subunits showed decreased association of cohesin with NIPBL (Fig. 5D, Additional file 2: Figure S6C). Of note, the NIPBL antibody is specific to the top band shown (Additional file 2: Figure S6D). While NIPBL does not co-purify with SMC3ac under WT conditions, it does strongly co-purify with PDS5A (Fig. 5D, Additional file 2: Figure S6C). This is surprising given reports that NIPBL and PDS5 proteins compete for interaction with RAD21 and do not occupy the same binding sites in yeast [4, 16, 17]. There is also a strong association of NIPBL with CTCF, which is surprising given that NIPBL is mainly localized to enhancers and promoters and not CTCF sites [48]. To determine if the decreased cohesin–NIPBL association affects NIPBL enrichment at enhancers, promoters, or CTCF sites, ChIP–qPCR was performed in WT siGLO and *Pds5a*^*−/−*^ siPds5b mESCs. Despite the decreased association of NIPBL with SMC3 following dual PDS5 loss, NIPBL levels were unchanged at representative enhancers, promoters, and CTCF sites (Fig. 5E). However, these results are consistent with reports that NIPBL can bind to chromatin without cohesin, and that bulk loading of cohesin onto chromatin is not affected upon loss of interaction with NIPBL [49–51].

The interaction between PDS5 and WAPL has been shown to be important for cohesin unloading during interphase [25, 26, 52]. Interestingly, immunoprecipitation of SMC3 following dual loss of PDS5 subunits showed only a slight decrease in the association of SMC3 with WAPL, not the total loss that would be expected if PDS5 proteins are the only interface by which WAPL associates with cohesin (Fig. 5D, Additional file 2: Figure S6C). These results support a prior report that WAPL can physically interact with cohesin subunits other than PDS5 [53]. Similar to NIPBL, ChIP–qPCR of WAPL in WT siGLO and *Pds5a*^*−/−*^ siPds5b mESCs revealed no differences in chromatin-bound WAPL levels across enhancers and promoters, though, a single significant decrease at a representative CTCF site (*p* < 0.0001) (Fig. 5F). Altogether, these results reveal that NIPBL–cohesin and WAPL–cohesin complexes can form in the absence of PDS5A and PDS5B.

## Discussion

Here we show for the first time that the mutually exclusive cohesin subunits PDS5A and PDS5B localize to shared binding sites across the genome of mammalian cells. Both PDS5A and PDS5B are strongly enriched at CTCF sites, and to a lesser extent at enhancers and promoters. The distribution of PDS5A and PDS5B is highly similar to that of the core cohesin complex member RAD21, as well as the mutually exclusive cohesin subunits STAG1 and STAG2 [12]. The strong overlap in PDS5A/B and STAG1/2 occupancy is unexpected given that STAG1/2 are associated with cohesin loading onto DNA and cohesin-mediated extrusion of DNA loops, while PDS5A/B inhibit cohesin loading and promote cohesin unloading from DNA in some contexts [3–6, 25, 26, 52]. This study determines the localization pattern of PDS5 proteins in the mammalian genome and tests the requirement for PDS5 proteins in cohesin occupancy and gene expression.

Whereas a single cohesin complex is thought to essentially always contain a STAG subunit, PDS5 interactions with the cohesin complex are proposed to be more transient. Co-immunoprecipitation studies revealed that PDS5A subunits can interact with both STAG1 and STAG2. Likewise, PDS5B subunits can interact with both STAG1 and STAG2, suggesting a lack of specificity for particular combinations of STAG and PDS5 subunits within cohesin complexes. Nevertheless, the interaction of PDS5B with the cohesin core subunit RAD21 appears to be weaker than the interaction of PDS5A with RAD21.

The two PDS5 proteins do not appear to compensate for each other, since loss of either PDS5A or PDS5B alone caused a slight, but not significant, increase in steady state protein levels of the remaining PDS5 subunit. In addition, the localization pattern and levels of the remaining PDS5 subunit were mostly unaffected upon siRNA depletion or knockout of a PDS5 protein, despite the variability and inconsistencies that these perturbations can produce. Furthermore, the genome-wide distribution of the core cohesin complex member RAD21 was not altered in *Pds5a*^*−/−*^*, Pds5b*^*−/−*^*,* or *Pds5a*^*−/−*^ siPds5b mESCs. This is consistent with a previous report that PDS5 depletion does not cause redistribution of RAD21 on the HeLa cell genome [37]. However, the retention of similar levels of cohesin on the genome in cells lacking PDS5 subunits is surprising, given the reported role of PDS5 proteins in WAPL-mediated unloading of cohesin from chromatin, the changes to genome organization following depletion of WAPL or PDS5 subunits, and changes to cohesin mobility following dual knockout of PDS5 subunits [20, 25, 26, 52, 54]. Furthermore, our results challenge the idea that the occupancy of PDS5 subunits at CTCF sites is critical for stopping extruding cohesin complexes, since we observe no change in cohesin levels at CTCF binding sites in cells lacking both PDS5A and PDS5B subunits. We did, however, observe that while PDS5B levels decreased at CTCF sites following cohesin depletion, PDS5A levels were unchanged in both WT siSMC3 mESCs and *Stag2*^*−/−*^ siStag1, conditions where most cohesin complexes are lost from the genome (seen here and in [12]). These results are consistent with a model where PDS5A, but not PDS5B, interacts with CTCF at insulator sites before the core cohesin complex arrives via DNA extrusion [19, 20]. Our results suggest that PDS5A and PDS5B are not specificity factors that dictate the pattern of cohesin localization across the genome. Taken together with our previous work, these results are consistent with a model, where variant cohesin complexes, with all possible combinations of STAG and PDS5 subunits, can exist in the cell and show similar distributions at CTCF sites, enhancers, and promoters, regardless of their subunit composition.

Several studies performed in yeast indicate that the loading factor Scc2 (NIPBL in mammals) and PDS5 are mutually exclusive subunits of cohesin complexes [4, 16, 17]. Cohesin–Scc2 (NIPBL) complexes can load onto chromosomes, hydrolyze ATP, and translocate efficiently, while cohesin–PDS5 molecules do not hydrolyze ATP or translocate and are released from chromosomes. Several studies suggest that the Scc2 (NIPBL) subunit is displaced from cohesin by the PDS5 subunit as cohesin extrusion stops, and that a cohesin complex cannot be bound by both NIPBL and PDS5 at the same time [4, 16, 17]. In mammalian cells, we find that PDS5A can copurify with NIPBL, suggesting that these two proteins interact. Furthermore, cohesin complexes that lack PDS5 subunits did not show increased interactions with NIPBL, consistent with a lack of competition between the PDS5 subunits and NIPBL for a single interface on cohesin. This result is consistent with a prior report and could indicate that the turnover of Scc2 (NIPBL) on chromatin may be too rapid to be affected by loss of PDS5 or too rapid to be detected in this type of assay [4]. A comprehensive look at the interaction of PDS5 with NIPBL, and the differences between the yeast and mammalian contexts, is needed to understand how these proteins differentially regulate cohesin function through a seemingly shared RAD21 (Scc1) interface.

Stable cohesin binding to the genome is promoted by the acetylation of the core cohesin member SMC3. Cohesin complexes with SMC3ac are enriched at CTCF sites and the anchors of DNA loops [32]. Depletion of CTCF strongly reduces SMC3ac, supporting the model that the CTCF–PDS5 interaction is important for SMC3 acetylation and stable cohesin residency [32, 43]. We observed that loss of PDS5 subunits under both acute conditions (WT siPds5a + siPds5b) and more chronic conditions (*Pds5a*^*−/−*^ siPds5b) lead to a decrease in the acetylated form of SMC3. Likewise, both acute (WT siStag1 + siStag2) and chronic (*Stag2*^*−/−*^ siStag1) loss of the STAG proteins lead to a similar decrease in the acetylated form of SMC3. The loss of SMC3ac in cells lacking STAG proteins appears to be due to decreased cohesin stability and binding to the genome, consistent with the reported role of STAG proteins in cohesin loading and translocating. The loss of SMC3ac in cells lacking PDS5 subunits is most likely due to decreased interactions between PDS5s and the acetyltransferases ESCO1/2, since loss of PDS5 proteins did not alter the proportion of cells in S phase or G2/M, consistent with previous results [37]. Cells lacking PDS5 subunits exhibit no change in chromatin-bound cohesin levels, corroborating previous results in mammalian SMC3ac mutants, suggesting that while PDS5 subunits can promote SMC3 acetylation, this may not directly impact cohesin loading and translocation [39]. Furthermore, while PDS5 subunits have been shown to contribute to cohesin unloading by facilitating the interaction of cohesin with WAPL [25, 26, 52], here we observe that loss of PDS5 subunits caused only a slight decrease in WAPL association with cohesin, consistent with a previous report that WAPL contacts additional cohesin subunits [53]. Together, these results support a model, where PDS5 subunits influence cohesin association with the genome by promoting both WAPL-mediated unloading of cohesin, as well as stable cohesin binding via SMC3 acetylation. Further studies should be aimed at understanding the complexities of this balance.

Our data suggest that cohesin rings lacking PDS5A or PDS5B subunits can still traverse the genome and localize to the appropriate target sites; however, they appear to display altered functions at those normal binding sites. Analysis of gene expression patterns shows that roughly half the genes that are differentially expressed upon loss of PDS5A are also misexpressed following loss of PDS5B. There are also large classes of genes that are only misexpressed upon loss of either PDS5A or PDS5B. Furthermore, cells lacking both PDS5A and PDS5B have more misexpressed genes than either single knockout, and a new class of genes is revealed that are only misexpressed when both PDS5 subunits are missing. The mechanism by which PDS5 proteins perform these partially independent roles in gene expression is not yet clear, and should be the subject of future studies. Importantly, the promoters of misexpressed genes were more likely to be engaged in a long-range cohesin-mediated interaction than promoters of genes not misexpressed following PDS5 perturbations. Therefore, while cohesin complexes display proper localization across the genome in cells lacking PDS5 subunits, it is possible that these common anchor sites are engaged in distinct DNA loops or have different properties, dynamics, or stabilities. Furthermore, despite the strong overlap between PDS5A/B and STAG1/2 binding to the genome, gene expression changes following dual loss of PDS5 subunits is not identical to the gene expression changes caused by dual loss of STAG subunits. While this work interrogated individual and combinatorial effects of PDS5A and PDS5B loss on cohesin localization and gene expression, and the relationships with STAG1 and STAG2, future studies are needed to determine how these four cohesin accessory proteins differentially influence cohesin dynamics and regulation of gene expression.

## Conclusions

This work utilizes isogenic mouse embryonic stem cells to interrogate the consequences of both acute and chronic loss of the HEAT repeat proteins, PDS5A and PDS5B. We find that PDS5A and PDS5B localize to shared binding sites across the genome, a pattern that overlaps that of other HEAT repeat proteins, STAG1 and STAG2. Individual and dual loss of PDS5 proteins does not cause mislocalization of cohesin on the genome, though does result in many gene expression changes. Despite the shared localization pattern of PDS5A, PDS5B, STAG1, and STAG2 subunits, many of the gene expression changes caused by dual loss of PDS5 subunits do not overlap changes caused by dual loss of STAG subunits. However, similar decreases in SMC3ac levels are observed upon dual loss of PDS5 subunits or STAG subunits. Whereas dual loss of STAG subunits decreases the level of chromatin-bound cohesin, dual loss of PDS5 subunits reduces cohesin association with NIPBL and WAPL.

## Methods

### Cell culture

Mouse embryonic stem cells (mESCs v6.5, male) were grown under standard ESC conditions as previously described [55], using KnockOut DMEM (Gibco, 10829-018) supplemented with 15% Fetal Bovine Serum (VWR, 97068-085). For ChIP-seq spike-in normalization, human embryonic kidney cells (HEK293T, female) were grown in DMEM (Gibco, 11995065) supplemented with 10% Bovine Calf Serum (Seradigm, 2100–500), 1X GlutaMAX (Thermo Fisher, 35050-061), 100 U/ml penicillin, and 100 ug/ml streptomycin (Thermo Fisher Scientific, 15140–122).

### Genome editing

WT mESCs were transfected with two plasmids using Lipofectamine 2000 (Thermo Fisher Scientific, 11-688-027), aiming to induce a small deletion within a single exon of the gene of interest. A unique sgRNA was cloned into a pX330 backbone containing SpCas9 and one of two fluorescent genes (eGFP or mCherry) (AddGene, 42230). One day post-transfection, 12,000 single cells were seeded on a CytoSort Array (Cell Microsystems, CS200S). Using a CellRaft AIR System (Cell Microsystems), cells fluorescent for both genes were collected, expanded, screened, and cryogenically stored. Sequences at the edit sites were determined following PCR of the region of interest and Sanger sequencing. Edit summaries and official allele name according to the International Committee on Standardized Genetic Nomenclature for Mice are shown below. Edit site sequences are depicted in Additional file 2: Figure S2A and guide RNA oligo sequences are listed in Additional file 5: Table S4. Isogenic *Stag2*^*−/−*^ knockout lines were previously generated and described [12].

*Pds5a*^*−/−*^ replicate 1 (also known as *Pds5a*^*em1Jdow*^) contains a heterozygous edit in exon 2 of *Pds5a* resulting in a homozygous knockout. Allele 1 has a 38 bp insertion and 18 bp deletion, while allele 2 has a 350 bp deletion that extends into the following intron. *Pds5a*^*−/−*^ replicate 2 (also known as *Pds5a*^*em2Jdow*^) contains a homozygous deletion of 176 bp in exon 2 of *Pds5a*. *Pds5b*^*−/−*^ replicate 1 (also known as *Pds5b*^*em1Jdow*^) contains a homozygous deletion of 5 bp in exon 3 of *Pds5b*. *Pds5b*^*−/−*^ replicate 2 (also known as *Pds5b*^*em2Jdow*^) contains a homozygous insertion of 1 bp in exon 3 of *Pds5b*.

### Chromatin immunoprecipitation (ChIP) and sequencing

mESCs were trypsinized (Gibco, 12604-013) and counted prior to crosslinking. Cells were crosslinked with 1% formaldehyde (Thermo Fisher Scientific, 28906) in PBS for 5 min then quenched with 2.5 M glycine. Crosslinked cells were lysed with 10 ml of cold Lysis Buffer 1 (50 mM Hepes–KOH pH7.5, 140 mM NaCl, 1 mM EDTA, 10% glycerol, 0.5% NP-40, and 0.25% Triton X-100) by rotating for 10 min at 4 ℃. After pelleting at 1350xg for 5 min, nuclei were lysed in 5 ml of room temperature Lysis Buffer 2 (10 mM Tris–HCl pH 8, 200 mM NaCl, 1 mM EDTA, and 0.5 mM EGTA) by rotating for 10 min at room temperature. After pelleting at 1350 × g for 5 min, supernatant removed and tubes were washed with 5 ml of cold shearing buffer (10 mM Tris pH 7.5, 1 mM EDTA, 0.1% SDS) and spun at 1350 × g for 5 min. Chromatin (pellet) was resuspended in 1 ml of shearing buffer and 5% of HEK293T chromatin, extracted using the same protocol, was added prior to sonication to be used later for spike-in normalization. All buffers were supplemented with 1X protease inhibitor cocktail (PIC) (Sigma Aldrich, 11697498001). Sonication of chromatin was performed using a Covaris E220 in milliTUBEs (Covaris, 520130) with the following settings: Duty Factor 5, PIP/W 140, and 200 cycles per burst for 12 min. Chromatin fragments of 200–1000 base pairs were generated. Following sonication, insoluble material was pelleted and removed by spinning samples for 10 min at 15,000 rpm at 4 ℃.

ChIPs were performed using the antibodies referred to in Additional file 1: Table S1. PDS5A and PDS5B antibodies were incubated with 50 ul Protein G Dynabeads (Invitrogen 10004D), using 3.5 ug and 4 ug of antibody, respectively. RAD21 antibody was incubated with 30 ul Protein G Dynabeads using 10 ug of antibody. Antibodies were incubated with beads for 6–8 h prior to the IP. Beads were washed three times with PBS with 1X protease inhibitor cocktail (PIC) (Sigma Aldrich, 11697498001) to remove unbound antibody prior to the addition of chromatin. The chromatin in shearing buffer was supplemented with NaCl and Triton X-100 to be in a ChIP buffer (20 mM Tris pH 7.5, 2 mM EDTA, 0.1% SDS, 150 mM NaCl, and 1% Triton X-100). Chromatin from 1 × 10^7^ cells was added to antibody conjugated beads and incubated rotating overnight at 4 °C. The next day, beads were washed with ChIP buffer, Wash Buffer 1 (20 mM Tris–HCl pH 8, 500 mM NaCl, 2 mM, EDTA, 0.1% SDS, and 1% Triton X-100), Wash Buffer 2 (10 mM Tris–HCl pH 8, 250 mM LiCl, 1 mM EDTA, and 1% NP-40), and Wash Buffer 3 (10 mM Tris pH 8, 1 mM EDTA, and 50 mM NaCl), each for 5 min rotating at 4 ℃. Chromatin was eluted from beads by adding 100 ul IP Elution Buffer (50 mM Tris pH 8, 10 mM EDTA, and 1% SDS) and incubating at 65 °C for 1 h, vortexing every 10 min. Supernatant was incubated at 65 °C overnight with addition of 5 ul Proteinase K (NEB, P8107S) to reverse crosslinks. The next day, DNA was purified using a ChIP DNA Clean and Concentrate kit (Zymo, D5205).

Libraries were prepared using the Kapa HyperPrep kit following manufacturer’s instructions (Roche/Kapa, KK8502). Sequencing was performed on either a HiSeq 4000 collecting 50 bp single-end reads (RAD21 in WT siGLO reps 1 and 2; RAD21 in *Pds5a*^*−/−*^ and *Pds5b*^*−/−*^ reps 1 and 2), or a NovaSeq 6000 SP collecting 50 bp paired-end reads (RAD21 in WT siPds5b, *Pds5a*^*−/−*^ siGLO, and *Pds5a*^*−/−*^ siPds5b reps 1 and 2; PDS5A in WT and *Pds5b*^*−/−*^ reps 1 and 2; PDS5B in WT and *Pds5a*^*−/−*^ reps 1 and 2).

### ChIP-seq analysis

ChIP-seq analysis was performed with a previously published custom script that can be found on GitHub: https://github.com/dowenlab and is described below [56]. Before processing, any sample that was split across two lanes of a sequencer had raw fastq files concatenated (files with “L1” and “L2”). If a sample was paired-end, only the first read (“R1”) was used for further analysis. Biological replicates were further concatenated as raw fastq files before alignment. Merged replicates were aligned to a merged genome containing both mouse genome assembly mm10 and human genome assembly hg38 using bowtie (v1.2.3) (parameters − v 2 − p 24 − S − m 1 –best –strata) [57]. Mchr was added as a prefix to mouse chromosomes for future distinction from human chromosomes. Duplicate sequences were removed using samtools (v1.11) markdup (− r − s) [58]. Respective mouse and human aligned reads were separated using samtools idxstats and counted with awk. A bam file containing only mouse reads was created using samtools view and then converted to bed format using bedtools (v2.29.0) bamtobed [59]. Reads were extended by 200 bp and extended bed files were used to call peaks using MACS (v2.2.7.1) with a false discovery rate of 1% (macs2 callpeak − f BED − g mm − q 0.01) [60]. Peak summits were expanded by 50 bp upstream and downstream and any expanded peaks overlapping a repeat element (defined using the Repeat Masker Track from UCSC genome browser) were removed using bedtools intersect (− v). A normalization factor was calculated for each ChIP-seq data set using the formula 1/h, where h is the number of human aligned reads in millions as described previously [61]. The bed file containing mouse reads was converted to a bedgraph file using bedtools genomecov (− bga − scale 1/h) before being converted to a bigwig file with bedGraphToBigWig from ucsctools (v320) [62]. *Z*-score normalization was performed where indicated using a custom R script from Spencer Nystrom of Dr. Daniel McKay’s lab.

Signal tracks for ChIP-seq data were visualized using IGV 2.4.10 desktop browser [63]. Average signal plots were generated using deeptools (v2.4.1) computeMatrix (reference-point for CTCF sites, promoters, enhancers, and denoted peak lists) followed by deeptools plotProfile [64]. Extended peak summits were used when performing peak overlaps with bedtools intersect. The list of promoters was obtained from UCSC transcription start sites. Enhancers were defined as sites co-occupied by the transcription factors OCT4, SOX2, and NANOG (ChIP-seq peaks) [65]. Any transcription start site bound by these factors was removed from the list of enhancers. “Other” sites are those remaining after taking the peak list of interest and removing sites that overlapped with CTCF sites, enhancers, and promoters using bedtools intersect (−v). Bar graphs with peak overlaps at functional sites were generated in Microsoft Excel. Heatmaps were generated using deeptools computeMatrix (reference-point) followed by deeptools plotHeatmap. Clustering of heatmaps was performed with k-means clustering as indicated in legends using deeptools plotHeatmap (–kmeans n). Union peak lists were made by concatenating respective peak lists and using bedtools merge to remove duplicate peaks (default overlap by 1 bp). Fingerprint plots were generated using deeptools plotFingerprint (–skipZeros). Correlation values were gathered from plots using deeptools multiBigwigSummary followed by plotCorrelation (–removeOutliers –skipZeros –corMethod Pearson). Differentially bound sites and corresponding MA plots were identified using DiffBind in R (v2.12.0) [44].

### ***ChIP***–***qPCR***

PDS5A ChIP (Bethyl, A300-089A) and PDS5B ChIP (Bethyl, A300-538A) were performed for two biological replicates each in WT siGLO, *Stag2*^*−/−*^ siStag1, and WT siSMC3 cells, and STAG1 ChIP and STAG2 ChIP was performed for two biological replicates each in WT siGLO and *Pds5a*^*−/−*^ siPds5b cells as described above. One negative control region and four CTCF sites were examined using primers listed in Additional file 5: Table S4. NIPBL ChIP (Santa Cruz, sc-374625) and WAPL ChIP (Bethyl, A300-268A) were also performed for two biological replicates each in WT siGLO and *Pds5a*^*−/−*^ siPds5b cells. NIPBL and WAPL ChIP samples were treated with two crosslinking reagents prior to sonication. Cells were incubated in PBS with 2 mM Di(N-succinimidyl) glutarate (DSG) (Sigma Aldrich, 80424), rotating at room temperature for 45 min prior to 1% formaldehyde crosslinking for 10 min. WAPL ChIP was performed using Protein A Dynabeads (Invitrogen, 10002D), while all others used Protein G. One negative control region and two each of enhancer, promoter, and CTCF sites were examined using the primers listed in Additional file 5: Table S4. Average fold change of ChIP enrichment was determined relative to the negative control region and 5% input material. Three technical replicates were performed for each of two biological replicates using PowerUp SYBR Green Master Mix (Applied Biosciences, A25742). Data represented as the mean average fold change ± the standard deviation of the six total replicates per ChIP. Significance between WT and depletion cell lines was determined for each primer set using a 2-tailed unpaired *t* test with asterisks indicating significance as depicted in figure legends.

### Co-immunoprecipitation (coIP)

Cells were collected via scraping in PBS. Samples for co-immunoprecipitation assays were extracted using a Nuclear Complex Co-IP Kit (Active Motif, 54001) with a homemade protocol for the nuclear fraction digestion step. Nuclei were isolated following manufacturer’s instructions. Nuclei were lysed in 200 ul Buffer A (10 mM HEPES pH 7.9, 10 mM KCl, 1.5 mM MgCl_2_, 340 mM sucrose, and 10% glycerol) with 1X protease inhibitor cocktail (PIC) (Sigma Aldrich, 11697498001) and digested with 10U of Benzonase (Sigma Aldrich, E1014) at 37 ℃ for 15 min. Reaction was quenched with 2 ul of 0.5 M EDTA and incubated on ice for 5 min. Samples spun at 5000 × g for 5 min at 4 °C, and supernatant containing nuclear proteins was collected. Protein levels were quantified using a Qubit Protein Assay quantification kit (Invitrogen, Q33211). 100 ug of protein was used in each IP under low and high stringency, native (uncrosslinked) conditions where indicated as per kit instructions. IP antibodies used include PDS5A (Bethyl, A300-089A), PDS5B (Bethyl, A300-538A), RAD21 (Abcam, ab992), SMC3 (Abcam, ab9263), NIPBL (Bethyl, A301-779A), and IgG (Bethyl, P120-101). Protein G Dynabeads (Invitrogen 10004D) were incubated with antibody for 4–6 h prior to addition of protein extracts. Clean-up of beads was performed following manufacturer’s instructions. IP material was eluted in 50 ul of IP Elution Buffer (50 mM Tris pH 8, 10 mM EDTA, and 1% SDS) at 65 °C for 1 h, vortexing every 10 min to keep beads in suspension.

### Fractionation

Cells were trypsinized (Gibco, 12604–013) and counted 48 h post-transfection of siRNA, collecting 10 million cells per condition. Chromatin-bound and unbound (nuclear soluble) fractions were collected using the Subcellular Protein Fractionation Kit for Cultured Cells (Thermo Scientific, 7884D). Collection of fractions was performed following manufacturer’s instructions for the 100ul packed cell volume, with additional PBS washes in between each collection.

### Western blotting

Confluent mESCs were washed with PBS and collected via scraping. Protein extracts were collected by resuspending cell pellets in 10 ml of Lysis Buffer A (10 mM HEPES pH 7.9, 10 mM KCl, 0.1 mM EDTA, and 0.1 mM EGTA) containing 1X protease inhibitor cocktail (PIC) (Sigma Aldrich, 11697498001) and rocking at 4 °C for 15 min. 1 ml of 10% NP-40 was added, samples immediately vortexed, and pelleted at 1350 × g for 5 min at 4 °C. Supernatant was removed and pellet was resuspended in 1 ml of cold Buffer TEN250/0.1 (50 mM Tris–HCl pH 7.5, 250 mM NaCl, 5 mM EDTA, and 0.1 mM NP-40) containing 1X PIC and rotated for a minimum of 30 min at 4 °C. After spinning samples at max speed for 10 min at 4 °C, the nuclear fraction (supernatant) was collected. Protein levels were quantified using a Qubit Protein Assay quantification kit (Invitrogen, Q33211), run in 4–20% Tris–Glycine gels (BioRad, 4568094), and transferred to PVDF membranes (VWR, BSP0161). Membranes were blocked for 1 h with 5% blocking grade buffer (BioRad, 1706404) and incubated overnight rocking at 4 °C with primary antibody. Primary antibodies used were PDS5A (Bethyl, A300-089A), PDS5B (Bethyl, A300-538A), RAD21 (Bethyl, A300-080A), SMC1A (Bethyl, A300-055A), SMC3 (Abcam, ab9263), STAG1 (Bethyl, A300-157A), STAG2 (Bethyl, A300-158A), CTCF (Active Motif, 61311), Histone H3 (Abcam, ab1791), Actin (Abcam, ab190476), SMC3ac (Millipore, MABE1073), NIPBL (Santa Cruz, sc-374625), and WAPL (Bethyl, A300-268A). All antibody washes were 3 × 10 min with 1X TBS-T. Secondary antibody incubations were for 1-h rocking at 4 °C. Secondary antibodies used were Donkey anti-Rabbit (GE Healthcare, NA934), Rabbit anti-Goat (Abcam, ab97100), and Goat anti-Mouse (Invitrogen, A16072). Membranes were imaged using either Thermo SuperSignal West Pico PLUS (34577) or Thermo SuperSignal West Femto (34094) chemiluminescent substrates with an Amersham Imager 600 (GE Healthcare). Quantification of blots were performed using Fiji [66]. Normalization of quantifications are described in figure legends where relevant.

### RNAi

Cells were counted and 5 × 10^5^ were plated per well in 6-well plates. 50 nM of siRNA or siGLO transfection control was transfected per well using DharmaFECT 1 transfection reagent (Dharmacon, T-2001) following manufacturer’s instructions. siRNA reagents used include siGLO (D-001630-01-0), siPds5b (M-058400-01-0005), siStag1 (M-041989-01-0005), siStag2 (M-057033-01-0005), siSMC3 (M-064492-01-0005), and siNipbl (M-048662-00-0005). Cells were harvested after 48 h for ChIP, protein extractions, RNA, or flow cytometry.

### RNA-sequencing and analysis

Three replicates of a single CRISPR clone were used for each genotype. Replicate one was used for *Pds5a*^*−/−*^ mESCs and replicate two was used for *Pds5b*^*−/−*^ mESCs. Cells were collected from 6-well plates 48-h after siRNA transfection. Cells were resuspended in Trizol (Invitrogen, 15596018) and RNA was extracted and purified using the Zymo Direct-zol RNA MiniPrep kit (Zymo, R2050). Libraries were prepared, with poly-A transcript enrichment, and sequenced by Novogene on a NovaSeq 6000 instrument with 150 bp paired end reads. RNA-sequencing samples are outlined in Additional file 1: Table S1.

Reads from RNA sequencing were aligned to the mm10 genome using STAR (v2.7.5) (–runThreadN 24-c SortedByCoordinate) [67]. Differentially expressed genes (DEGs) were identified using DESeq2 (v1.24.0) (padj < 0.01) [45]. Overlaps of DEGs and all union DEG lists were generated in R using dplyr (v1.0.8) [68]. Correlation plots were generated using GraphPad PRISM followed by Pearson correlation analysis. Heatmaps of log2 fold changes in gene expression were generated in R using pheatmap (RRID:SCR_016418). Bar graphs of log2 fold change in expression for cell identity genes were made using Microsoft Excel with significance determined by DESeq2. For gene ontology (GO) analysis, the lists of *Pds5a*^*−/−*^ specific, *Pds5b*^*−/−*^ specific, Common, and Redundant differentially expressed genes were subset into upregulated and downregulated gene sets. GO analysis was performed on each of these eight gene sets using the ShinyGO software package (FDR < 0.05) [69]. The same was done for the eight subsets of DEGs that were *Pds5a*^*−/−*^ siPds5b specific, *Stag2*^*−/−*^ siStag1 specific, and common to both conditions (Additional file 2: Figure S4I). The top 30 enriched terms for each subset are presented in Additional file 4: Table S3. Relative protein abundance of PDS5A and PDS5B (log10 normalized iBAQ intensity) in 123 cell and tissue types was downloaded from ProteomicsDB [47]. Gene expression counts from 1,019 transformed human cell lines were downloaded from the Cancer Cell Line Encyclopedia. Expression of PDS5 proteins was represented as a ratio of Pds5a to Pds5b transcript levels, and plotted as a cumulative distribution function using Microsoft Excel. Read counts from Pds5a and Pds5b transcripts for mESCs were determined from our own RNA-seq data in WT cells using the normalized read output from DESeq2. Significance between transcripts levels in WT and knockout genotypes was determined using a 2-tailed unpaired *t* test. Violin plots were generated using GraphPad Prism. Significance was determined using a Kruskal–Wallis test followed by Dunn’s multiple comparisons test. Lists of Super-enhancer Domains, Polycomb Domains, and ChIA–PET anchors were obtained from [46], with coordinates converted from mm9 to mm10 using UCSC LiftOver. The non-DEG promoters correspond to genes not differentially expressed in each of the PDS5 perturbation conditions, and genes were excluded if no transcripts were detected in any of the conditions.

### Cell cycle analysis

For flow cytometry, 48 h post siRNA transfection mESCs were incubated with 10um of EdU (Santa Cruz, sc-284628) at 37° C for 30 min. Cells were collected following our normal trypsinization protocol. After washing with PBS, cells were spun for 3 min at 2300 × g, resuspended in 500 ul of 4% paraformaldehyde in PBS and incubated for 15 min at room temperature. 1 ml of 1% BSA was added to aid in pelleting the cells, and then cells were resuspended in 1 ml of 1% BSA + 0.5% Triton X-100 and incubated for another 15 min at room temperature. After pelleting at 2300 × g for 5 min, cells were resuspended in 500 ul of labeling solution (PBS, 1 mM CuSO_4_, 1 uM AlexaFluor 647 (Life Technologies, A10277), and 100 mM ascorbic acid) and incubated at room temperature for 30 min protected from light. Cells had 1 ml of 1% BSA + 0.5% Triton X-100 added before spinning at 2300 × g for 5 min. Cells were then resuspended in 500 ul of DAPI staining solution (1% BSA + 0.5% Triton X-100, 100ug/ml RNAse, 1 ug/ml DAPI (Life Technologies, D1306)) and incubated at 37° C for 1 h protected from light. 1 ml of PBS was added to cells before spinning for 5 min at 2300 × g. Cells resuspended in a final volume of 300ul PBS, passed through a filter top tube, and taken for flow analysis. Cells were sorted using an Attune NxT flow cytometer and cell cycle analysis was performed using FlowJo™ v10.8 Software (BD Life Sciences). Data represented as the average ± the standard deviation of three biological replicates. Significance between WT and depletion conditions was determined for each phase using a 2-tailed unpaired *t* test with asterisks indicating significance as depicted in figure legends.

Proliferation assays of the same seven conditions used in flow cytometry were performed as previously described with three biological replicates [12]. From this, the population doubling time between 72 and 84 h was calculated for cells in each condition (Population Doubling Time = Time in culture/ PD, where PD = log2(# cells at harvest /# cells originally plated)). Together with the percentage of cells in each cell cycle phase, the length of time in hours per cell cycle phase was calculated. Significance between WT and depletion conditions was determined for each phase using a 2-tailed unpaired *t* test with asterisks indicating significance as depicted in figure legends.

## Supplementary Information

**Additional file1: Table S1**: Summary table of the data sets used in this study including antibody product number, genotypes, factor accession numbers, and background accession numbers.

**Additional file2**: Supplementary figures and corresponding figure legends (S1, S2, S3, S4, S5, and S6).

**Additional file3: Table S2**: Full lists of differentially expressed genes for all conditions used in RNA-seq analysis in this study. Samples are compared to either WT siGLO or *Pds5a *^*-/-*^ siGLO conditions as indicated.

**Additional file4: Table S3**: Lists of the top 30 GO-terms for both up- and down-regulated genes in the gene classes indicated.

**Additional file5: Table S4**: Oligos used in this study and the assay they were used in are indicated.

## Data Availability

The data sets supporting the conclusions of this article are available in the GEO repository under accession number GSE199356. Data can be accessed by going to https://www.ncbi.nlm.nih.gov/geo/query/acc.cgi?acc=GSE199356. All individual accession numbers are available in Additional file 1: Table S1.

## Abbreviations

mESC: Mouse embryonic stem cell
WT: Wildtype
ChIP-seq: Chromatin immunoprecipitation followed by sequencing
coIP: Co-immunoprecipitation
IP: Immunoprecipitation
RNA-seq: RNA sequencing
DEGs: Differentially expressed genes
SD: Super-enhancer domain
PD: Polycomb domain
GO: Gene ontology
