# Supplementary material for: PDS5A and PDS5B differentially affect gene expression without altering cohesin localization across the genome
Source: Epigenetics Chromatin. 2022 Aug 19;15:30. doi: 10.1186/s13072-022-00463-6 (PMC9392266; doi:10.1186/s13072-022-00463-6)
Supplement: Supplementary file 1 — Additional file1: Table S1: Summary table of the data sets used in this study including antibody product number, genotypes, factor accession numbers, and background accession numbers. [file 13072_2022_463_MOESM1_ESM.pdf]

Table S1. Summary and accession numbers of data used in this study

Data Type	Factor	Antibody	Cell Type	Factor Accession Number	Background Accession Number
ChIP-seq	PDS5A	Bethyl A300-089A	WT mESC	GSM5970922<br>GSM5970923	GSE199356
	PDS5B	Bethyl A300-538A	WT mESC	GSM5970924<br>GSM5970925	GSE199356
	RAD21	Bethyl A300-080A	WT mESC	GSM4074281<br>GSM4074282	GSE137285
	CTCF	Active Motif 61311	WT mESC	GSM4074279<br>GSM4074280	GSE137285
	OCT4	Santa Cruz sc-8628X	WT mESC	GSM1082340	GSE44286
	SOX2	R&D Systems MAB2018	WT mESC	GSM1082341	GSE44286
	NANOG	Bethyl A300-397A	WT mESC	GSM1082342	GSE44286
	RAD21	Abcam ab992	WT mESC	GSM4280484<br>GSM4280485	GSE144116
	PDS5A	Bethyl A300-089A	*Pds5b* $^{-/-}$ mESC	GSM5970926<br>GSM5970927	GSE199356
	RAD21	Abcam ab992	*Pds5b* $^{-/-}$ mESC	GSM5970928<br>GSM5970929	GSE199356
	PDS5B	Bethyl A300-538A	*Pds5a* $^{-/-}$ mESC	GSM5970930<br>GSM5970931	GSE199356
	RAD21	Abcam ab992	*Pds5a* $^{-/-}$ mESC	GSM5970932<br>GSM5970933	GSE199356
	RAD21	Abcam ab992	WT siGLO mESC	GSM5970934<br>GSM5970935	GSE199356
			WT siPds5b mESC	GSM5970936<br>GSM5970937	GSE199356
			Pds5a $^{-/-}$ siGLO mESC	GSM5970938<br>GSM5970939	GSE199356
			Pds5a $^{-/-}$ siPds5b mESC	GSM5970940<br>GSM5970941	GSE199356
	STAG1	Bethyl A300-0157A	WT mESC	GSM4280480<br>GSM4280481	GSE144116
	STAG2	Bethyl A300-0159A	WT mESC	GSM4280482<br>GSM4280483	GSE144116
RNA-seq	-	-	WT siGLO mESC	GSM5968203<br>GSM5968204<br>GSM5968205	GSE199356
	-	-	*Pds5a* $^{-/-}$ siGLO mESC	GSM5968209<br>GSM5968210<br>GSM5968211	GSE199356
	-	-	*Pds5b* $^{-/-}$ siGLO mESC	GSM5968212<br>GSM5968213<br>GSM5968214	GSE199356
			WT siPds5b mESC	GSM5968206<br>GSM5968207<br>GSM5968208	GSE199356
	-	-	*Pds5a* $^{-/-}$ siPds5b mESC	GSM5968215<br>GSM5968216<br>GSM5968217	GSE199356
	-	-	*Pds5a* $^{-/-}$ siStag1 mESC	GSM5968218<br>GSM5968219<br>GSM5968220	GSE199356
	-	-	*Pds5a* $^{-/-}$ siStag2 mESC	GSM5968221<br>GSM5968222<br>GSM5968223	GSE199356
	-	-	WT siGLO mESC	GSM4280519<br>GSM4280520<br>GSM4280521	GSE144116
	-	-	*Stag2* $^{-/-}$ siStag1 mESC	GSM4280531<br>GSM4280532<br>GSM4280533	GSE144116
