# Supplementary material for: PDS5A and PDS5B differentially affect gene expression without altering cohesin localization across the genome
Source: Epigenetics Chromatin. 2022 Aug 19;15:30. doi: 10.1186/s13072-022-00463-6 (PMC9392266; doi:10.1186/s13072-022-00463-6)
Supplement: Supplementary file 2 — Additional file2: Supplementary figures and corresponding figure legends (S1, S2, S3, S4, S5, and S6). [file 13072_2022_463_MOESM2_ESM.pdf]

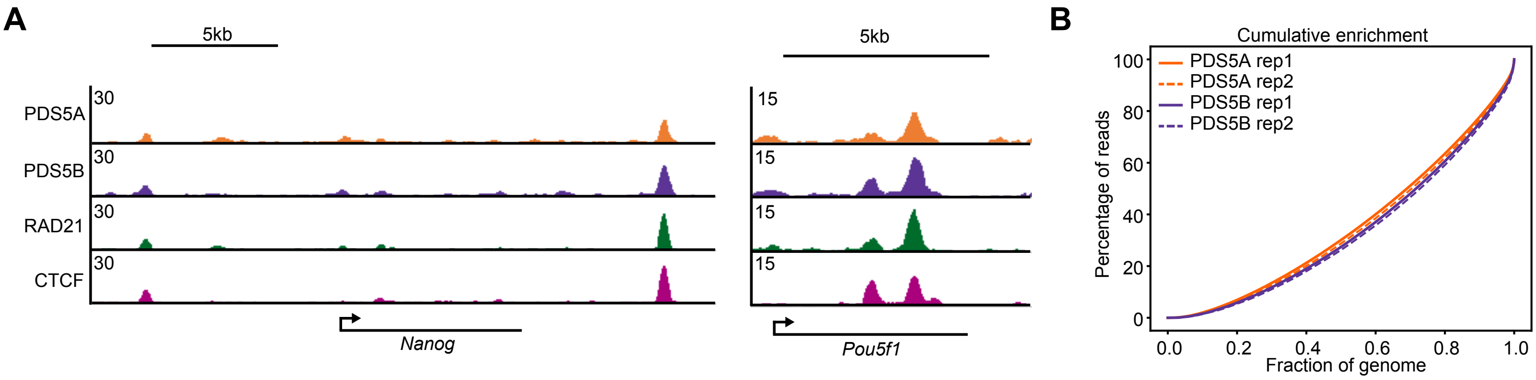

Figure S1. PDS5A and PDS5B occupy the same sites across the genome

A. Genome browser tracks of PDS5A, PDS5B, RAD21, and CTCF ChIP-seq signal near the *Nanog* and *Pou5f1*(OCT4) genes in WT mESCs (*Z*-score normalized).

B. Fingerprint plot showing cumulative enrichment of reads per fraction of the genome for each biological replicate of PDS5A and PDS5B ChIP-seq data.

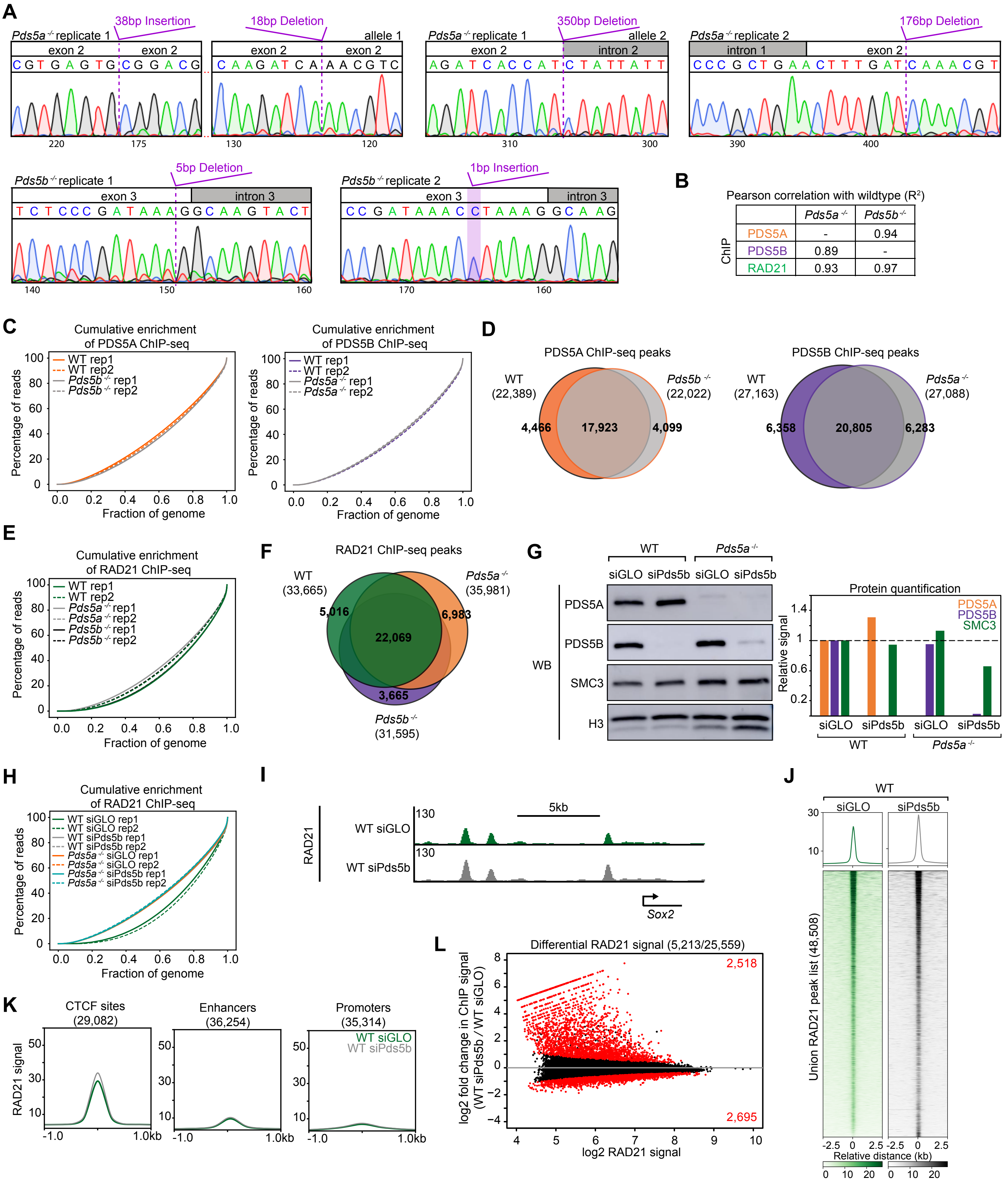
A
Pds5a -/- replicate 1
38bp Insertion
exon 2
exon 2
CGTGAGTGCGGACG
18bp Deletion
allele 1
exon 2
exon 2
CAAGATCAAACGTC
220
175
130
120
Pds5a -/- replicate 1
350bp Deletion
allele 2
exon 2
intron 2
AGATCACCATCTATTATT
310
300
Pds5a -/- replicate 2
176bp Deletion
intron 1
exon 2
CCCGCTGAACTTTGATCAAACGT
390
400
Pds5b -/- replicate 1
5bp Deletion
exon 3
intron 3
TCTCCCGATAAAGGCAAGTACT
140
150
160
Pds5b -/- replicate 2
1bp Insertion
exon 3
intron 3
CCGATAAACCTAAAGGCAAG
170
160
B
Pearson correlation with wildtype (R2)
ChIP
Pds5a -/-
Pds5b -/-
PDS5A
-
0.94
PDS5B
0.89
-
RAD21
0.93
0.97
C
Cumulative enrichment of PDS5A ChIP-seq
WT rep1
WT rep2
Pds5b -/- rep1
Pds5b -/- rep2
Percentage of reads
Fraction of genome
Cumulative enrichment of PDS5B ChIP-seq
WT rep1
WT rep2
Pds5a -/- rep1
Pds5a -/- rep2
Percentage of reads
Fraction of genome
D
PDS5A ChIP-seq peaks
WT (22,389)
Pds5b -/- (22,022)
4,466
17,923
4,099
PDS5B ChIP-seq peaks
WT (27,163)
Pds5a -/- (27,088)
6,358
20,805
6,283
E
Cumulative enrichment of RAD21 ChIP-seq
WT rep1
WT rep2
Pds5a -/- rep1
Pds5a -/- rep2
Pds5b -/- rep1
Pds5b -/- rep2
Percentage of reads
Fraction of genome
F
RAD21 ChIP-seq peaks
WT (33,665)
Pds5a -/- (35,981)
5,016
22,069
6,983
3,665
Pds5b -/- (31,595)
G
WT
Pds5a -/-
siGLO
siPds5b
siGLO
siPds5b
WB
PDS5A
PDS5B
SMC3
H3
Protein quantification
PDS5A
PDS5B
SMC3
Relative signal
siGLO
siPds5b
siGLO
siPds5b
WT
Pds5a -/-
H
Cumulative enrichment of RAD21 ChIP-seq
WT siGLO rep1
WT siGLO rep2
WT siPds5b rep1
WT siPds5b rep2
Pds5a -/- siGLO rep1
Pds5a -/- siGLO rep2
Pds5a -/- siPds5b rep1
Pds5a -/- siPds5b rep2
Percentage of reads
Fraction of genome
I
RAD21
WT siGLO
WT siPds5b
130
130
5kb
Sox2
J
WT
siGLO
siPds5b
Union RAD21 peak list (48,508)
Relative distance (kb)
K
CTCF sites (29,082)
Enhancers (36,254)
Promoters (35,314)
WT siGLO
WT siPds5b
RAD21 signal
-1.0
1.0kb
L
Differential RAD21 signal (5,213/25,559)
2,518
2,695
log2 fold change in ChIP signal (WT siPds5b / WT siGLO)
log2 RAD21 signal

Figure S2. Cohesin localization is largely independent of PDS5 proteins

A. Sequencing chromatograms for *Pds5a* $^{-/-}$ and *Pds5b* $^{-/-}$ mESCs. Purple dashed lines indicate edit sites.
B. Pearson correlation (R^2) values for PDS5A, PDS5B, and RAD21 ChIP-seq signal between WT and knockout mESCs.
C. Fingerprint plots showing cumulative enrichment of reads per fraction of the genome for each biological replicate of PDS5A and PDS5B ChIP-seq data in WT and knockout mESCs.
D. Overlap of PDS5A ChIP-seq peaks in WT and *Pds5b* $^{-/-}$ mESCs as well as overlap of PDS5B ChIP-seq peaks in WT and *Pds5a* $^{-/-}$ mESCs.
E. Fingerprint plot showing cumulative enrichment of reads per fraction of the genome for each biological replicate of RAD21 ChIP-seq in WT, *Pds5a* $^{-/-}$, and *Pds5b* $^{-/-}$ mESCs.
F. Overlap of RAD21 ChIP-seq peaks in WT, *Pds5a* $^{-/-}$, and *Pds5b* $^{-/-}$ mESCs.
G. Western blot analysis and quantification of nuclear lysates of WT siGLO, WT siPds5b, *Pds5a* $^{-/-}$ siGLO, and *Pds5a* $^{-/-}$ siPds5b mESCs. Quantifications are relative to H3 loading control.
H. Fingerprint plot showing cumulative enrichment of reads per fraction of the genome for each biological replicate of RAD21 ChIP-seq in WT siGLO, WT siPds5b, *Pds5a* $^{-/-}$ siGLO, and *Pds5a* $^{-/-}$ siPds5b mESCs.
I. Genome browser tracks of RAD21 ChIP-seq signal in WT siGLO and WT siPds5b mESCs at the *Sox2* gene locus.
J. Heatmaps of RAD21 ChIP-seq signal in WT siGLO and WT siPds5b mESCs at a union peak list of all RAD21 peaks in both conditions.
K. Average signal plots of RAD21 ChIP-seq in WT siGLO and WT siPds5b mESCs at CTCF sites, enhancers, and promoters.
L. MA plot showing differential RAD21 ChIP-seq signal between WT siPds5b and WT siGLO mESCs at conserved binding sites.

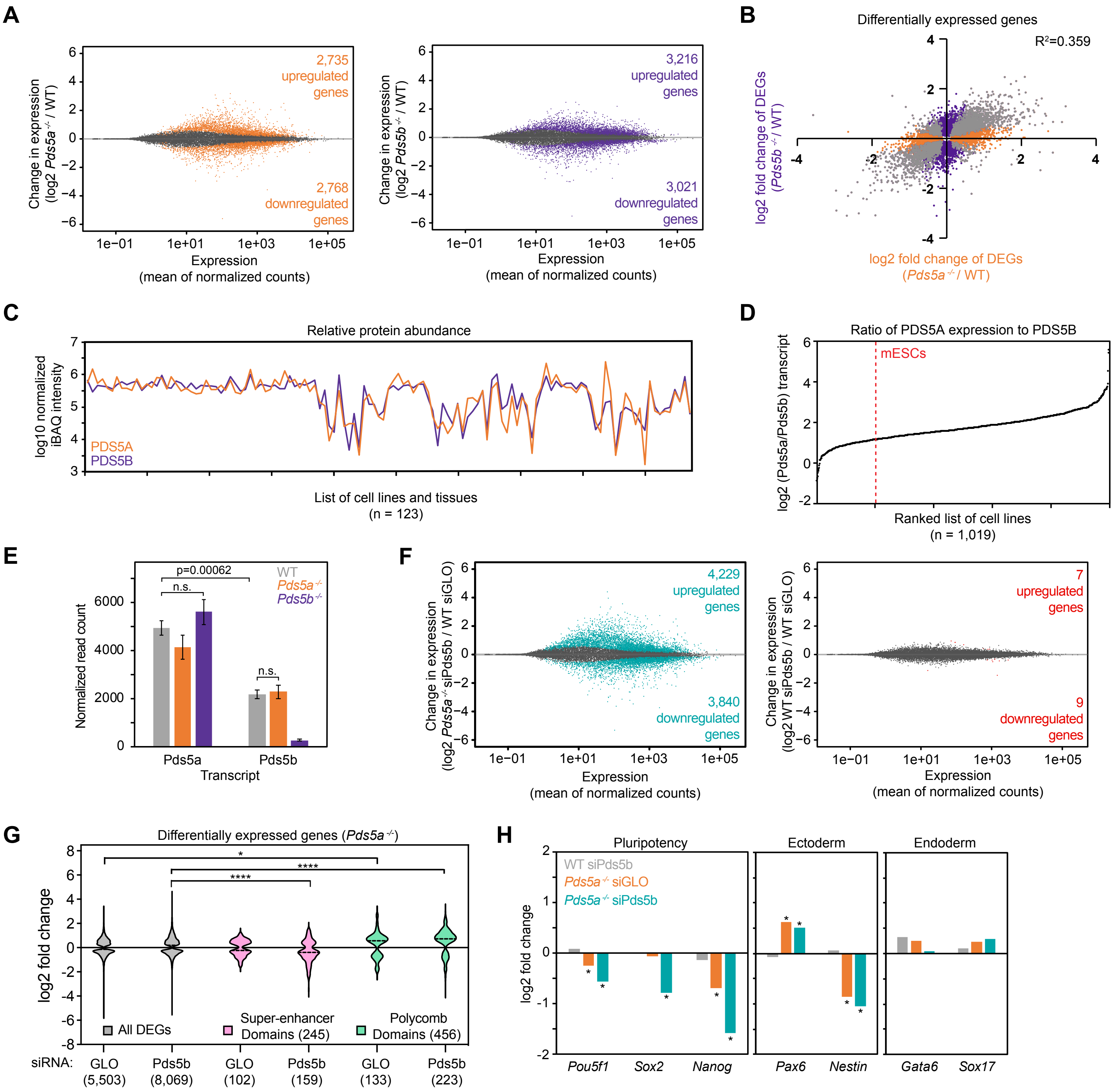
A
Change in expression
(log2 Pds5a-/- / WT)
2,735
upregulated
genes
2,768
downregulated
genes
Expression
(mean of normalized counts)
Change in expression
(log2 Pds5b-/- / WT)
3,216
upregulated
genes
3,021
downregulated
genes
Expression
(mean of normalized counts)
B
Differentially expressed genes
R²=0.359
log2 fold change of DEGs
(Pds5b-/- / WT)
log2 fold change of DEGs
(Pds5a-/- / WT)
C
Relative protein abundance
log10 normalized
iBAQ intensity
PDS5A
PDS5B
List of cell lines and tissues
(n = 123)
D
Ratio of PDS5A expression to PDS5B
log2 (Pds5a/Pds5b) transcript
mESCs
Ranked list of cell lines
(n = 1,019)
E
Normalized read count
p=0.00062
n.s.
n.s.
WT
Pds5a-/-
Pds5b-/-
Pds5a
Pds5b
Transcript
F
Change in expression
(log2 Pds5a-/- siPds5b / WT siGLO)
4,229
upregulated
genes
3,840
downregulated
genes
Expression
(mean of normalized counts)
Change in expression
(log2 WT siPds5b / WT siGLO)
7
upregulated
genes
9
downregulated
genes
Expression
(mean of normalized counts)
G
Differentially expressed genes (Pds5a-/-)
log2 fold change
All DEGs
Super-enhancer
Domains (245)
Polycomb
Domains (456)
siRNA:
GLO
(5,503)
Pds5b
(8,069)
GLO
(102)
Pds5b
(159)
GLO
(133)
Pds5b
(223)
H
Pluripotency
Ectoderm
Endoderm
WT siPds5b
Pds5a-/- siGLO
Pds5a-/- siPds5b
log2 fold change
Pou5f1
Sox2
Nanog
Pax6
Nestin
Gata6
Sox17

Figure S3. Role of PDS5A and PDS5B in gene expression

A. MA plots showing DEGs in *Pds5a* $^{-/-}$ or *Pds5b* $^{-/-}$ mESCs relative to WT mESCs. All cells were treated with siGLO as a transfection control.

B. Correlation plot of log2 fold changes of all DEGs in *Pds5a* $^{-/-}$ and *Pds5b* $^{-/-}$ mESCs. The DEGs commonly misexpressed in both conditions are in gray while those specific to *Pds5a* $^{-/-}$ mESCs or *Pds5b* $^{-/-}$ mESCs are in orange and purple, respectively.

C. Relative abundance of PDS5A and PDS5B (log10 normalized iBAQ intensity) in 123 cell and tissue types from ProteomicsDB (Lautenbacher et al. 2022).

D. Relative transcript levels (log2 Pds5a/Pds5b) in our mESCs (raw ratio value = 2.27) and 1,019 cell lines from the Cancer Cell Line Encyclopedia.

E. Normalized read counts for Pds5a and Pds5b transcripts in WT, *Pds5a* $^{-/-}$ and *Pds5b* $^{-/-}$ mESCs. Data represented as the average ± standard deviation across three biological replicates. A 2-tailed unpaired t-test was used to determine significance between groups with n.s. representing no significance.

F. MA plots showing DEGs in *Pds5a* $^{-/-}$ siPds5b or WT siPds5b mESCs relative to WT siGLO mESCs.

G. Violin plot of log2 fold changes for all DEGs, those within Super-enhancer Domains, and those within Polycomb Domains for *Pds5a* $^{-/-}$ siGLO, and *Pds5a* $^{-/-}$ siPds5b mESCs. Significance was determined using a Kruskal-Wallis test followed by Dunn's multiple comparisons test. Asterisks indicate significant differences between groups (* $p < 0.05$, **** $p < 0.0001$).

H. Bar graphs of log2 fold change in expression of pluripotency genes (*Pou5f1, Sox2, Nanog*), ectodermal lineage genes (*Pax6 and Nestin*), and endodermal lineage genes (*Gata6 and Sox17*) in WT siPds5b, *Pds5a* $^{-/-}$ siGLO, and *Pds5a* $^{-/-}$ siPds5b mESCs. Asterisks indicate significant differences from WT siGLO mESCs determined using DESeq2 (padj < 0.01).

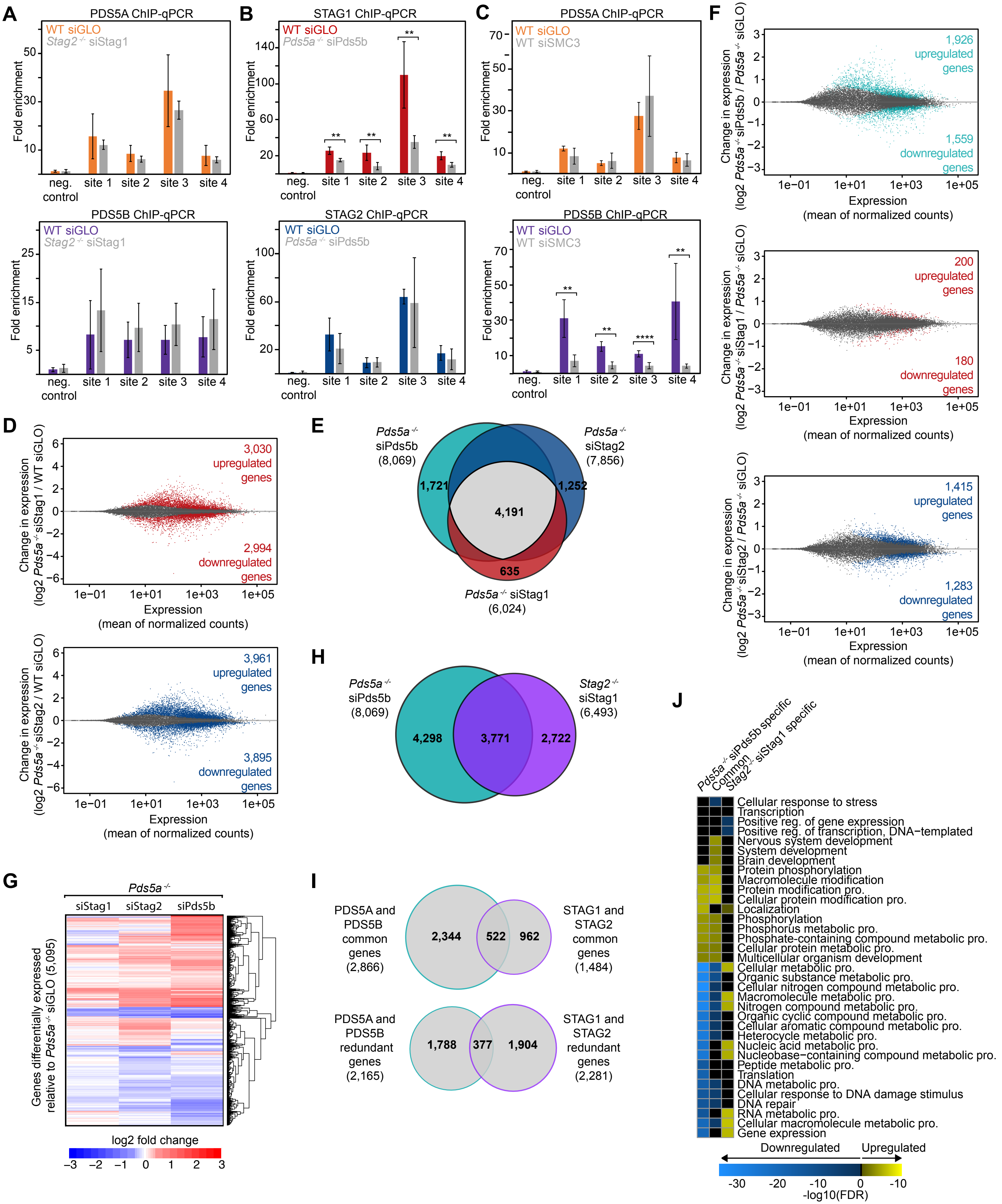
A
PDS5A ChIP-qPCR
WT siGLO
Stag2-/- siStag1
Fold enrichment
neg. control
site 1
site 2
site 3
site 4
PDS5B ChIP-qPCR
B
STAG1 ChIP-qPCR
WT siGLO
Pds5a-/- siPds5b
STAG2 ChIP-qPCR
C
PDS5A ChIP-qPCR
WT siGLO
WT siSMC3
PDS5B ChIP-qPCR
F
1,926 upregulated genes
1,559 downregulated genes
Change in expression (log2 Pds5a-/- siPds5b / Pds5a-/- siGLO)
Expression (mean of normalized counts)
200 upregulated genes
180 downregulated genes
Change in expression (log2 Pds5a-/- siStag1 / Pds5a-/- siGLO)
1,415 upregulated genes
1,283 downregulated genes
Change in expression (log2 Pds5a-/- siStag2 / Pds5a-/- siGLO)
D
3,030 upregulated genes
2,994 downregulated genes
Change in expression (log2 Pds5a-/- siStag1 / WT siGLO)
3,961 upregulated genes
3,895 downregulated genes
Change in expression (log2 Pds5a-/- siStag2 / WT siGLO)
E
Pds5a-/- siPds5b (8,069)
Pds5a-/- siStag2 (7,856)
Pds5a-/- siStag1 (6,024)
1,721
1,252
4,191
635
H
Pds5a-/- siPds5b (8,069)
Stag2-/- siStag1 (6,493)
4,298
3,771
2,722
G
Pds5a-/-
siStag1
siStag2
siPds5b
Genes differentially expressed relative to Pds5a-/- siGLO (5,095)
log2 fold change
I
PDS5A and PDS5B common genes (2,866)
2,344
522
962
STAG1 and STAG2 common genes (1,484)
PDS5A and PDS5B redundant genes (2,165)
1,788
377
1,904
STAG1 and STAG2 redundant genes (2,281)
J
Pds5a-/- siPds5b specific
Common
Stag2-/- siStag1 specific
Cellular response to stress
Transcription
Positive reg. of gene expression
Positive reg. of transcription, DNA−templated
Nervous system development
System development
Brain development
Protein phosphorylation
Macromolecule modification
Protein modification pro.
Cellular protein modification pro.
Localization
Phosphorylation
Phosphorus metabolic pro.
Phosphate-containing compound metabolic pro.
Cellular protein metabolic pro.
Multicellular organism development
Cellular metabolic pro.
Organic substance metabolic pro.
Cellular nitrogen compound metabolic pro.
Macromolecule metabolic pro.
Nitrogen compound metabolic pro.
Organic cyclic compound metabolic pro.
Cellular aromatic compound metabolic pro.
Heterocycle metabolic pro.
Nucleic acid metabolic pro.
Nucleobase−containing compound metabolic pro.
Peptide metabolic pro.
Translation
DNA metabolic pro.
Cellular response to DNA damage stimulus
DNA repair
RNA metabolic pro.
Cellular macromolecule metabolic pro.
Gene expression
Downregulated
Upregulated
-log10(FDR)

Figure S4. Knockdown of STAG1 and STAG2 in cells lacking PDS5A causes changes similar to loss of PDS5B in *Pds5a*$^{-/-}$ mESCs

A. ChIP-qPCR for PDS5A and PDS5B in WT siGLO and *Stag2*$^{-/-}$ siStag1 mESCs. Fold enrichment at four different CTCF sites, relative to 5% input material and the negative control region is depicted. Data represented as mean ± standard deviation across two biological replicates, each with three technical replicates. A 2-tailed unpaired t-test was used to determine significance between WT and dual depletion lines for every primer set. No significance was found for any of the primer sets.
B. ChIP-qPCR for STAG1 and STAG2 in WT siGLO and *Pds5a*$^{-/-}$ siPds5b mESCs. Fold enrichment at four different CTCF sites, relative to 5% input material and the negative control region is depicted. Data represented as mean ± standard deviation across two biological replicates, each with three technical replicates. A 2-tailed unpaired t-test was used to determine significance between WT and dual depletion lines for every primer set. Asterisks indicate significant differences between groups (** $p < 0.01$), otherwise, no significance was found.
C. ChIP-qPCR for PDS5A and PDS5B in WT siGLO and WT siSMC3 mESCs. Fold enrichment at four different CTCF sites, relative to 5% input material and the negative control region is depicted. Data represented as mean ± standard deviation across two biological replicates, each with three technical replicates. A 2-tailed unpaired t-test was used to determine significance between WT and depletion lines for every primer set. Asterisks indicate significant differences between groups (**** $p < 0.0001$, ** $p < 0.01$), otherwise, no significance was found.
D. MA plots showing DEGs in *Pds5a*$^{-/-}$ siStag1 or *Pds5a*$^{-/-}$ siStag2 mESCs relative to WT siGLO mESCs.
E. Overlap of differentially expressed genes (DEGs) in *Pds5a*$^{-/-}$ siPds5b, *Pds5a*$^{-/-}$ siStag1, and *Pds5a*$^{-/-}$ siStag2 mESCs relative to WT siGLO mESCs.
F. MA plots showing DEGs in *Pds5a*$^{-/-}$ siStag1, *Pds5a*$^{-/-}$ siStag2, and *Pds5a*$^{-/-}$ siPds5b mESCs relative to *Pds5a*$^{-/-}$ siGLO mESCs.
G. Clustered heatmap of log2 fold changes for a combined list of DEGs in *Pds5a*$^{-/-}$ siStag1, *Pds5a*$^{-/-}$ siStag2, and *Pds5a*$^{-/-}$ siPds5b mESCs relative to *Pds5a*$^{-/-}$ siGLO mESCs.
H. Overlap of DEGs in *Pds5a*$^{-/-}$ siPds5b and *Stag2*$^{-/-}$ siStag1 mESCs relative to WT siGLO mESCs (Arruda et al., 2020).
I. Overlaps of DEGs of Common and Redundant genes respectively from *Pds5a*$^{-/-}$ siPds5b and *Stag2*$^{-/-}$ siStag1 mESCs relative to WT siGLO mESCs (Arruda et al., 2020).
J. Gene Ontology (GO) terms for biological processes that are *Pds5a*$^{-/-}$ siPds5b specific, *Stag2*$^{-/-}$ siStag1 specific, and common to both conditions (from overlap in S4H) (Arruda et al., 2020).

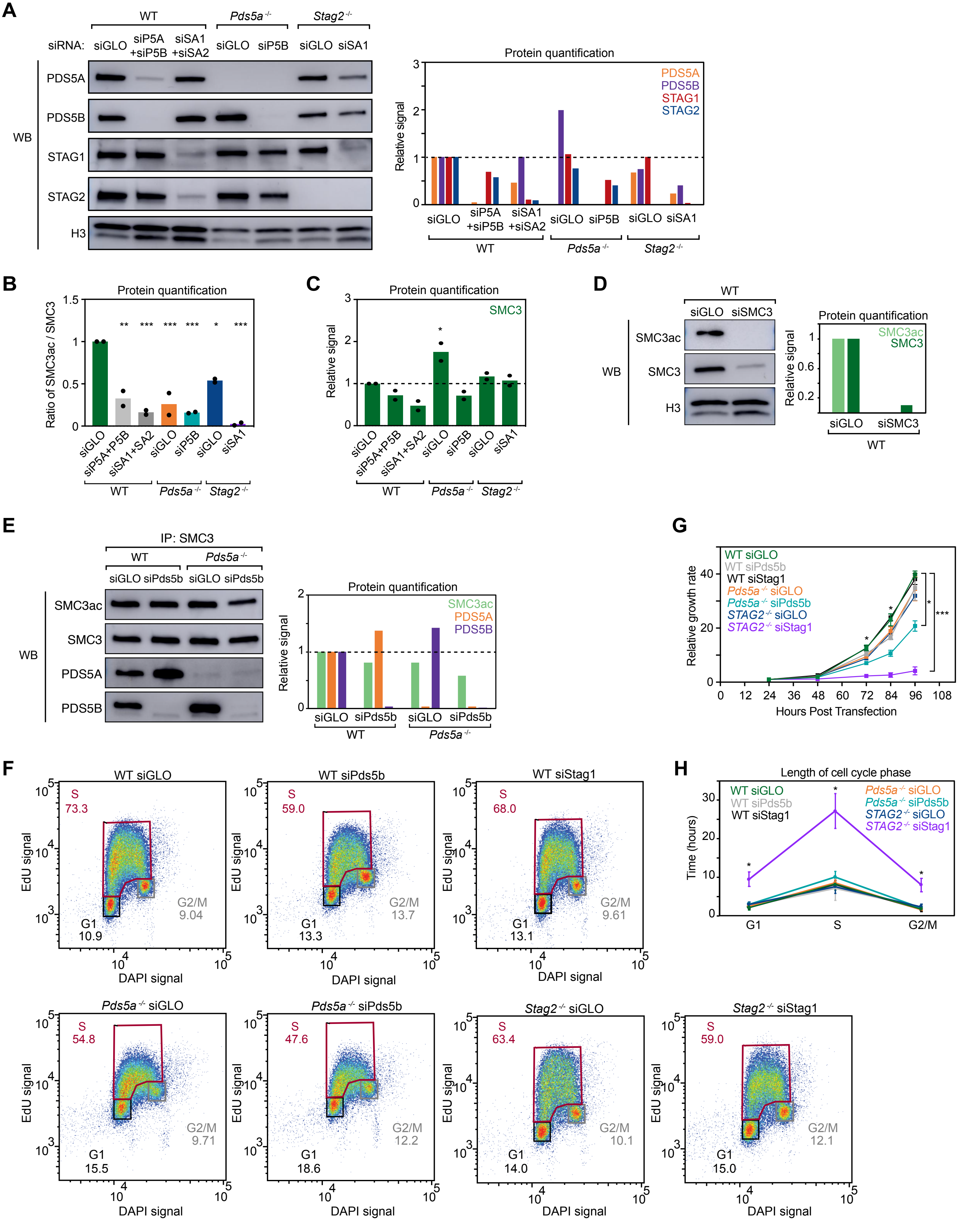
A
WT
Pds5a-/-
Stag2-/-
siRNA: siGLO siP5A +siP5B siSA1 +siSA2 siGLO siP5B siGLO siSA1
WB
PDS5A
PDS5B
STAG1
STAG2
H3
Protein quantification
Relative signal
PDS5A
PDS5B
STAG1
STAG2
siGLO siP5A +siP5B siSA1 +siSA2 siGLO siP5B siGLO siSA1
WT Pds5a-/- Stag2-/-
B
Protein quantification
Ratio of SMC3ac / SMC3
** *** *** *** * ***
siGLO siP5A+P5B siSA1+SA2 siGLO siP5B siGLO siSA1
WT Pds5a-/- Stag2-/-
C
Protein quantification
SMC3
Relative signal
siGLO siP5A+P5B siSA1+SA2 siGLO siP5B siGLO siSA1
WT Pds5a-/- Stag2-/-
D
WT
siGLO siSMC3
WB
SMC3ac
SMC3
H3
Protein quantification
SMC3ac
SMC3
Relative signal
siGLO siSMC3
WT
E
IP: SMC3
WT Pds5a-/-
siGLO siPds5b siGLO siPds5b
WB
SMC3ac
SMC3
PDS5A
PDS5B
Protein quantification
SMC3ac
PDS5A
PDS5B
Relative signal
siGLO siPds5b siGLO siPds5b
WT Pds5a-/-
G
WT siGLO
WT siPds5b
WT siStag1
Pds5a-/- siGLO
Pds5a-/- siPds5b
STAG2-/- siGLO
STAG2-/- siStag1
Relative growth rate
Hours Post Transfection
F
WT siGLO
S 73.3
G1 10.9
G2/M 9.04
WT siPds5b
S 59.0
G1 13.3
G2/M 13.7
WT siStag1
S 68.0
G1 13.1
G2/M 9.61
Pds5a-/- siGLO
S 54.8
G1 15.5
G2/M 9.71
Pds5a-/- siPds5b
S 47.6
G1 18.6
G2/M 12.2
Stag2-/- siGLO
S 63.4
G1 14.0
G2/M 10.1
Stag2-/- siStag1
S 59.0
G1 15.0
G2/M 12.1
EdU signal
DAPI signal
H
Length of cell cycle phase
WT siGLO
WT siPds5b
WT siStag1
Pds5a-/- siGLO
Pds5a-/- siPds5b
STAG2-/- siGLO
STAG2-/- siStag1
Time (hours)
G1 S G2/M

Figure S5. Acute and chronic loss of cohesin accessory proteins reduces SMC3ac levels

A. Western blot analysis and quantification of nuclear lysates showing knockdown efficiencies in the seven siRNA conditions. Quantifications are relative to the average of SMC3 and two H3 loading controls.
B. Quantification of Figure 5A depicted as the ratio of SMC3ac signal over SMC3 signal for each condition from two biological replicates. Significance was determined using a one-way ANOVA followed by Tukey's multiple comparisons test. Asterisks indicate significant differences from WT siGLO mESCs (***$p < 0.001$, **$p < 0.01$, *$p < 0.05$).
C. Quantification of Figure 5A showing total SMC3 signal from two biological replicates. Significance was determined using a one-way ANOVA followed by Tukey's multiple comparisons test. Asterisk indicates significant differences from WT siGLO mESCs (*$p = 0.026$). Quantifications are relative to H3 loading control.
D. Western blot analysis and quantification of SMC3ac and SMC3 in WT siGLO and WT siSMC3 mESCs. Quantifications are relative to H3 loading control.
E. Western blot analysis and quantification following co-immunoprecipitation of SMC3 done in the presence of 5mM sodium butyrate, in WT siGLO, WT siPds5b, *Pds5a*$^{-/-}$ siGLO, and *Pds5a*$^{-/-}$ siPds5b mESCs. Quantifications are relative to SMC3 signal.
F. Scatterplots showing flow cytometry results for one of three biological replicates for all seven conditions quantified in Figure 5B.
G. Proliferation rate of mESC lines represented relative to the original plating density. Data represented as the average of three biological replicates ± standard deviation. A two-way ANOVA followed by Tukey's multiple comparisons test was used to determine significance. Asterisks indicate significant differences from WT siGLO mESCs (*$p < 0.05$, ***$p < 0.001$). Significance at 72 and 84hr is for *Stag2*$^{-/-}$ siStag1 relative to WT siGLO mESCs.
H. Length of cell cycle phases calculated from the population doubling time (the 72 and 84hr timepoints in the proliferation assay from Figure S5G) and the percentage of cells in each cell cycle phase from the flow cytometry analysis. A 2-tailed unpaired t-test was used to determine significance. Asterisks indicate significant differences from WT siGLO mESCs (*$p < 0.05$).

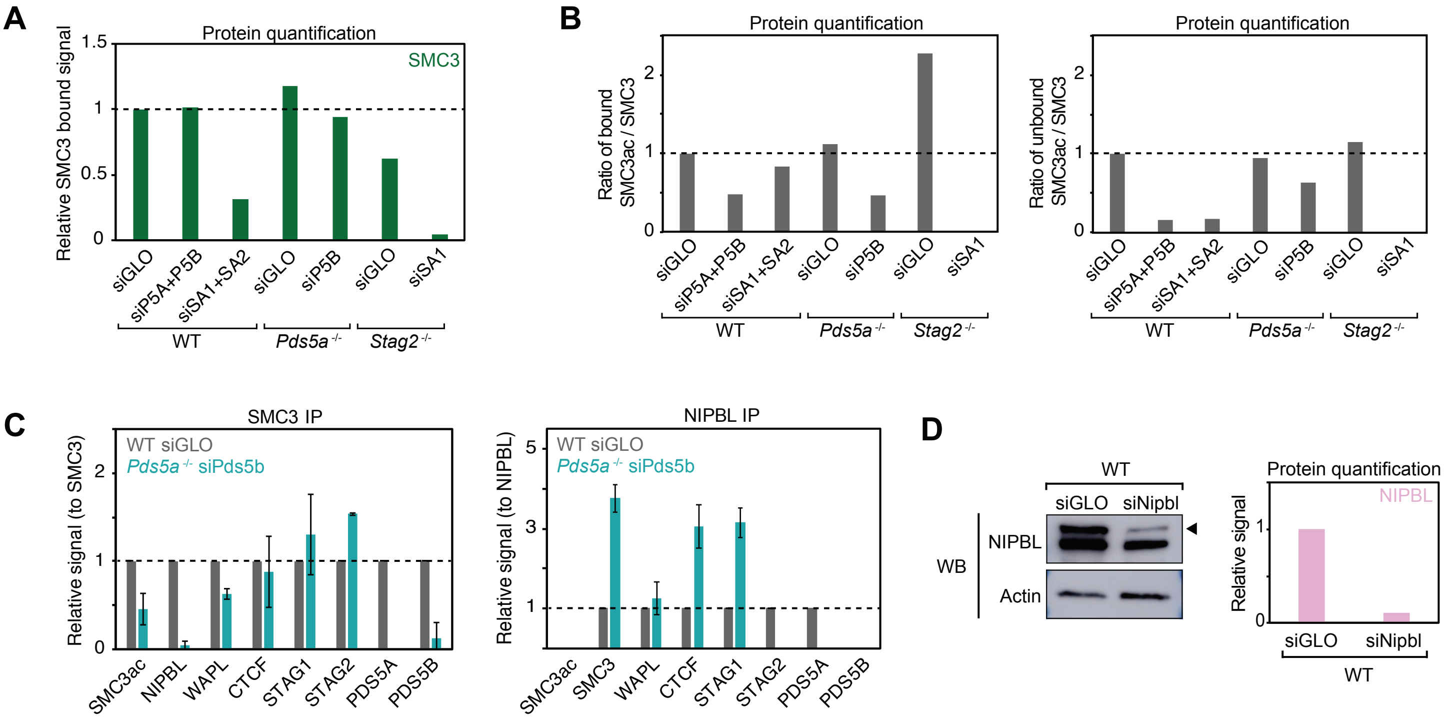

Figure S6. Loss of cohesin accessory proteins differentially affect bound cohesin

A. Quantification of Figure 5B showing chromatin-bound SMC3 signal in all seven siRNA conditions all relative to WT siGLO. Quantifications are relative to H3 loading control.
B. Quantification of Figure 5B depicted as the ratio of SMC3ac signal over SMC3 signal for both chromatin-bound and unbound fractions in all seven siRNA conditions all relative to WT siGLO.
C. Quantification of Figure 5C for both the SMC3 IP relative to SMC3 signal, and the NIPBL IP relative to NIPBL signal. Data represented as mean ± standard deviation across two biological replicates.
D. Western blot analysis and quantification of NIPBL in WT siGLO and WT siNipbl mESCs to test antibody specificity. Black triangle in NIPBL blot points to the specific band (top). Quantifications are relative to Actin loading control.
